# Transcriptional Changes in Pulmonary Phagocyte Subsets Dictate the Outcome Following Interaction With The Fungal Pathogen *Cryptococcus neoformans*

**DOI:** 10.3389/fimmu.2021.722500

**Published:** 2021-09-28

**Authors:** Ashlee N. Hawkins, Brenden F. Determann, Benjamin N. Nelson, Karen L. Wozniak

**Affiliations:** Department of Microbiology and Molecular Genetics, Oklahoma State University, Stillwater, OK, United States

**Keywords:** *Cryptococcus*, pulmonary phagocytes, macrophage subsets, dendritic cell subsets, pulmonary innate immunity

## Abstract

With over 220,000 cases and 180,000 deaths annually, *Cryptococcus neoformans* is the most common cause of fungal meningitis and a leading cause of death in HIV/AIDS patients in Sub-Saharan Africa. Either *C. neoformans* can be killed by innate airway phagocytes, or it can survive intracellularly. Pulmonary murine macrophage and dendritic cell (DC) subsets have been identified in the naïve lung, and we hypothesize that each subset has different interactions with *C. neoformans*. For these studies, we purified murine pulmonary macrophage and DC subsets from naïve mice – alveolar macrophages, Ly6c^-^ and Ly6c^+^ monocyte-like macrophages, interstitial macrophages, CD11b^+^ and CD103^+^ DCs. With each subset, we examined cryptococcal association (binding/internalization), fungicidal activity, intracellular fungal morphology, cytokine secretion and transcriptional profiling in an *ex vivo* model using these pulmonary phagocyte subsets. Results showed that all subsets associate with *C. neoformans*, but only female Ly6c^-^ monocyte-like macrophages significantly inhibited growth, while male CD11b^+^ DCs significantly enhanced fungal growth. In addition, cytokine analysis revealed that some subsets from female mice produced increased amounts of cytokines compared to their counterparts in male mice following exposure to *C. neoformans*. In addition, although cells were analyzed *ex vivo* without the influence of the lung microenviroment, we did not find evidence of phagocyte polarization following incubation with *C. neoformans*. Imaging flow cytometry showed differing ratios of cryptococcal morphologies, c-shaped or budding, depending on phagocyte subset. RNA sequencing analysis revealed the up- and down-regulation of many genes, from immunological pathways (including differential regulation of MHC class I in the antigen processing pathway and the cell adhesion pathway) and pathways relating to relating to metabolic activity (genes in the Cytochrome P450 family, genes related to actin binding, calcium voltage channels, serine proteases, and phospholipases). Future studies gaining a more in-depth understanding on the functionality of individual genes and pathways specific to permissive and non-permissive pulmonary phagocytes will allow identification of key targets when developing therapeutic strategies to prevent cryptococcal meningitis.

## Introduction

The fungal pathogen *Cryptococcus neoformans* is typically acquired by inhalation from the environment [reviewed in (1–3)] and initially interacts with innate phagocytes in the lung, since these cells serve as the first line of defense against invading airway pathogens (4–8). Indeed, several studies point to an important role for innate cells such as dendritic cells (DCs) and macrophages in initial stages of cryptococcal control (9–15). During infection, pulmonary macrophages and dendritic cells (DCs) are the first innate phagocytes to interact with *C. neoformans*, and they are both able to phagocytose the pathogen (16) [reviewed in (17)]. In our previous studies as well as in other studies in the literature, human and murine DCs and their products kill or inhibit the growth of *C. neoformans* (13, 18, 19), while human and animal macrophages can either kill the fungus (20–24) or allow intracellular replication of the organism, depending upon the experimental model [reviewed in (25–28)] (24, 29–34). The dogma in the cryptococcal field is that *Cryptococcus* can replicate within macrophages in the lung, and then macrophages containing cryptococci can traffic to the brain, releasing the intracellular cryptococcal cells to cause meningitis (35–38). However, the specific macrophage and/or DC subset(s) involved in permissive intracellular growth & transport is unknown.

The naïve (uninfected) murine pulmonary cavity consists of a heterogeneous population of macrophages and DC populations (39–42). In fact, tissue resident macrophages are derived from diverse progenitors, including embryonic origins (fetal liver and fetal yolk sac) (43) [reviewed in (44, 45)], while other macrophage and DC subsets are terminally differentiated from the hematopoietic lineage (46–50). These subsets have been identified by flow cytometry and/or gene expression profiling as well as by lineage tracing (43, 45, 48, 51–59). In naïve murine lungs, the distinct populations of pulmonary macrophages include: alveolar macrophages, interstitial macrophages, monocyte-like Ly6c^+^ macrophages and monocyte-like Ly6c^-^ macrophages, and the distinct populations of conventional pulmonary DCs include CD11b^+^ DCs and CD103^+^ DCs, each expressing unique cell surface markers (55, 56) [reviewed in (60)].

As previously mentioned, macrophages containing intracellular cryptococci are thought to be the primary mechanism by which *C. neoformans* traffics from the lung to the brain to cause meningitis (35–38). Depletion of alveolar macrophages in a murine model of infection using clodronate liposomes significantly reduced the number of cryptococci in the brain, and reduced death (32). However, clodronate treatment is known to deplete all phagocytes (macrophages, DCs and even neutrophils) (61), so these results may not be specific to only alveolar macrophages. Indeed, alveolar macrophages are not considered to be migratory, although some studies have shown that they can migrate to the draining lymph nodes (62). *C. neoformans* can survive intracellularly in macrophages by producing many factors (including laccase, phospholipase B, and HSP70) and by detoxification of copper, interference with host signaling pathways, actin flashes, induction of lysosomal damage, induction of host cell apoptosis and leaky phagosomes [reviewed in (25, 26, 63, 64)]. However, the majority of these studies have been performed in the J774A.1 cell line, which is not representative of mouse or human primary macrophages (65). Additional studies have used mouse bone marrow derived macrophages or human PBMC-derived macrophages (34, 66, 67), but none have used pulmonary macrophage or DC subsets to identify the specific subset(s) responsible for transport from the lung to the brain or to identify mechanisms responsible for killing *vs* intracellular survival in these individual pulmonary phagocyte subsets. We were interested in identifying the pulmonary macrophage and DC subsets responsible for permissive intracellular growth and those responsible for fungicidal activity. Furthermore, we were interested in identifying the genes and signaling pathways in each subset that correlated with each response.

Subsets of pulmonary phagocytes not only exist, but these different populations can have different interactions with the same infectious pathogen (68). Recent data from mouse pulmonary macrophage subsets following interaction with *Mycobacterium tuberculosis* (Mtb) have shown that macrophages derived from different lineages have different metabolic profiles, which contributes to their ability to either kill or intracellularly harbor Mtb (69, 70). Based on this we hypothesized that pulmonary phagocyte subsets would also interact differently with *C. neoformans*, and these differences in antifungal activity may be due to differentially expressed genes and signal transduction pathways activated or repressed in each subset following interaction (contact and/or uptake) with *C. neoformans*.

In the cryptococcal literature thus far, few studies have examined individual macrophage and DC subset interactions with *C. neoformans*. In one study using a glucosylceramide deficient cryptococcal strain, Δ*gcs1*, use of clodronate liposomes in mice deficient in NK cells and T cells increased survival and decreased cryptococcal dissemination to the brain, suggesting that alveolar macrophages were involved in cryptococcal dissemination (36). However, as mentioned earlier, clodronate liposomes are not alveolar macrophage-specific, so this more likely shows that a phagocyte population (in general) is responsible for cryptococcal dissemination. One study examining interstitial macrophages showed that interstitial macrophages harbored intracellular *C. neoformans* following intratracheal administration (37), and transfer of infected cells into recipient mice *via* the tail vein led to hematogenous dissemination of *C. neoformans* to the brain (37). While the monocyte-like macrophage populations have not been specifically examined, inflammatory monocytes (typically defined as Ly6c^hi^/CCR2^+^) (71) may be important in cryptococcal clearance. In CCR2^-/-^ mice infection with *C. neoformans* leads to Th2-type cytokine responses, increased pulmonary fungal burden, and decreased pulmonary infiltration of macrophages and DCs (15, 72–75). Indeed, Ly6c^hi^ CCR2^+^ monocytes are recruited to the lung during cryptococcal infection and have been shown to differentiate into antifungal macrophages and DCs (73, 75). In somewhat conflicting studies, depletion of inflammatory monocytes using the CCR2 DTR system results in improved survival, reduced dissemination, and a decrease in pulmonary fungal burden (76). The inflammatory monocytes upregulate genes involved in M2 macrophage polarization, suggesting that inflammatory monocytes can differentiate into M2 macrophages, which is detrimental to the host response to cryptococcal infection (76).

Dendritic cells (DCs) also play an important role in controlling *Cryptococcus* infections [reviewed in (77)]. DCs are recruited to the murine lung during a cryptococcal infection (16), and in a protective model of cryptococcal infection, increased numbers of DCs are recruited to the lungs in protected mice compared to non-protected mice, suggesting an anti-cryptococcal role for DCs (78). Following DC-cryptococcal interactions, DCs mediate the adaptive immune response by presentation of antigen to *Cryptococcus*-specific T cells (16). The depletion of pulmonary DCs, *via* the administration of diphtheria toxin (DT) to transgenic (Tg) mice, leads to increased morbidity and mortality as well increased B cell and neutrophil accumulation which causes severe lung inflammation (15).

Currently, there are no published studies that provide an in-depth analysis of individual pulmonary phagocyte subsets and their initial interactions with *C. neoformans.* Thus, the overall objective of this study was to provide a detailed look into this interaction using an *ex-vivo* system with an aim of determining whether pulmonary phagocyte subsets interact differently with *C. neoformans* and to identify potential factors leading to these differences. Our data show that the different pulmonary phagocyte subsets, when cultured *ex-vivo* with *C. neoformans*, do interact differently with *C. neoformans* and these differences are sex-dependent. In addition, we have identified specific genes and signaling pathways potentially associated with these responses.

## Materials And Methods

### Strains and Media

*C. neoformans* strains H99 (Serotype A, mating type α) and mCherry producing strain KN99mCH (serotype A, KN99 mating type α), a kind gift from Dr. Jennifer Lodge (Washington University, St. Louis, MO), were recovered from 15% glycerol stocks, stored at -80°C prior to use in this study. Strains were maintained on yeast peptone dextrose (YPD) media agar plates. Yeast cells were grown for 18h at 30°C while shaking in YPD broth and collected by centrifugation. After incubation, the cells were washed three times with sterile phosphate-buffered saline (PBS), and viable yeast quantified using trypan blue dye exclusion on a hemocytometer before being diluted to the appropriate concentration for each particular assay.

### Mice

Male and female BALB/c mice, approximately 5-6 weeks of age (Charles River Laboratories, Wilmington, MA) were used throughout these studies and were housed at Oklahoma State University Animal Resources. All studies were approved by Oklahoma State University’s Institutional Animal Care and Use Committee (IACUC), and mice were handled according to IACUC guidelines.

### Alveolar Macrophage Collection

Prior to euthanasia, mice were intravenously injected with anti-CD45 FITC (25ug/ml) (BD Biosciences, Franklin Lakes, NJ) diluted in PBS without calcium and magnesium 4-5 minutes prior to sacrifice in order to tag intravascular leukocytes within systemic circulation and separate these from pulmonary extravascular mononuclear phagocytes (79). Mice were then euthanized *via* CO_2_ inhalation followed by cervical dislocation. After euthanizing, the mouse trachea was exposed by making a vertical midline incision. A sterile 18-gauge cannula was inserted horizontally within the trachea with an attached syringe containing 1 ml sterile ice-cold PBS + 0.5% EDTA. The lungs were then lavaged three times with this solution to collect the bronchoalveolar lavage fluid (BAL), which was pooled into a sterile tube kept on ice.

### Pulmonary Tissue Macrophage and Dendritic Cell Collection

For collection of lung tissue macrophages and dendritic cells, following collection of BAL, lungs were removed using aseptic technique, minced, and placed into digestion buffer (RPMI with 0.1mg/ml collagenase type IV (Sigma-Aldrich, St. Louis, MO). Tissues were enzymatically digested at 37°C for 30 min in 10 ml of digestion buffer, as previously described (11, 16, 78). Following incubation, the digested tissues were then successively filtered through sterile 70- and 40-μm nylon filters (BD Biosciences) to enrich for leukocytes, and then cells were washed with sterile Hank’s Balanced Salt Solution (HBSS). Erythrocytes were lysed by incubation in RBC lysis buffer (eBioscience, San Diego, CA) for 3 min on ice followed by a 2-fold excess of PBS.

### Purification of Pulmonary Macrophage and Dendritic Cell Subsets

**Figure 1:**
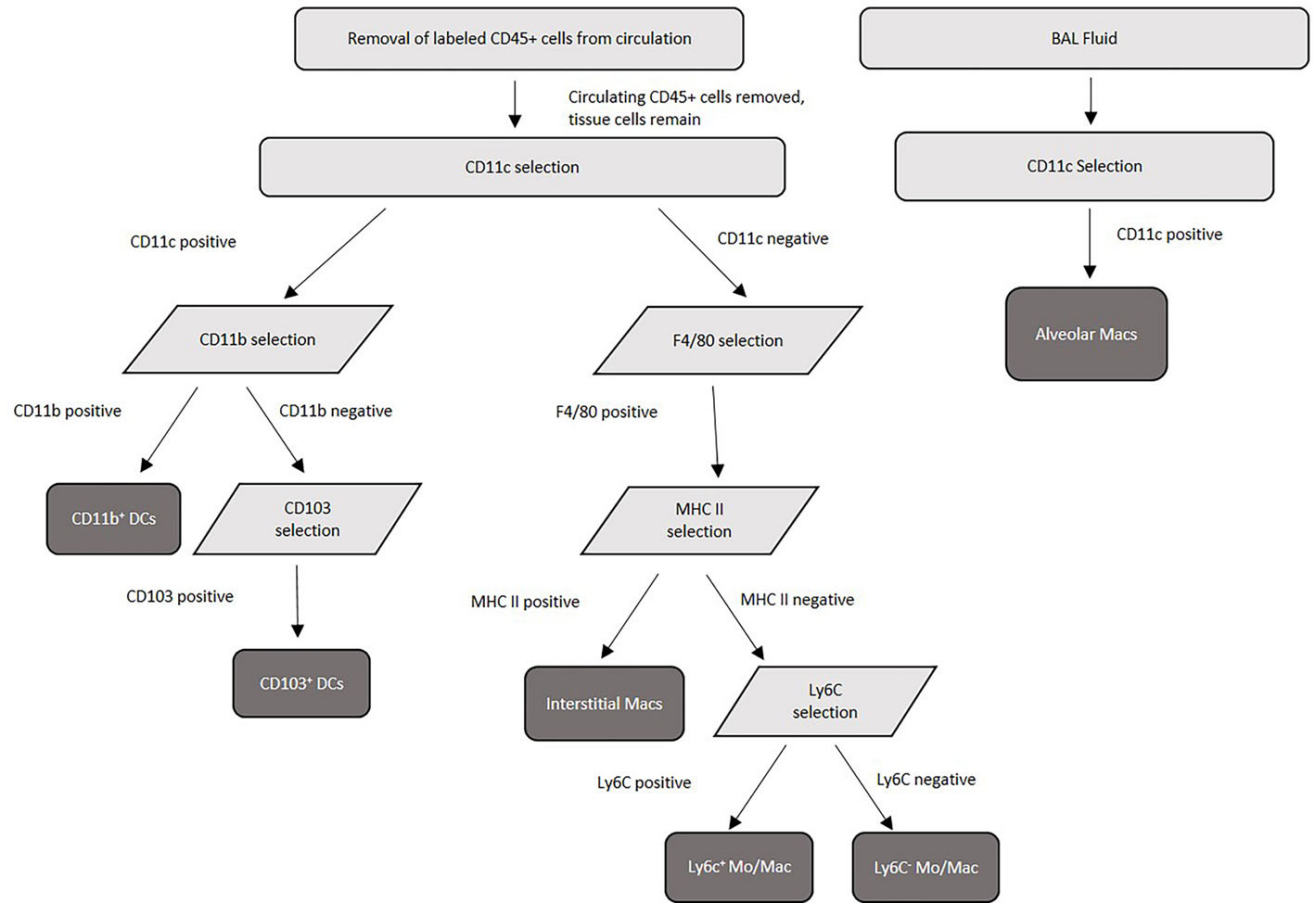
Purification scheme for murine pulmonary tissue macrophage and dendritic cell subsets. Lung tissue was pooled from 20 mice/experiment, collagenase digested, then separated into single-cell suspensions before magnetic separation. Following lung tissue collagenase digestion, labeled FITC-CD45^+^ cells from the circulation were removed by anti-FITC magnetic separation, and tissue macrophage and dendritic cell subsets were separated using a series of surface antibody-specific magnetic separations. Cell purity was verified by flow cytometry post-separation.

Alveolar macrophages were purified from a single cell suspension of BAL. Cells were enriched for alveolar macrophages first by blocking Fc receptors using purified rat anti-mouse CD16/CD32 (Mouse BD Fc block) (BD Biosciences) followed by positive selection using anti-CD11c microbeads according to manufacturer’s instructions (Miltenyi Biotec, Auburn, CA). Purity was verified using Labeling Check Reagent conjugated to VioBlue (Miltenyi Biotec) and CD11c and F4/80 fluorescently labeled antibodies by flow cytometry flow cytometry using an Acea Novocyte flow cytometer (Agilent Technologies, Santa Clara, CA), and purity above 95% was routinely achieved. For purification of pulmonary tissue macrophages (interstitial macrophages, Ly6c^+^ monocyte-like macrophages, and Ly6c^-^ monocyte-like macrophages) and tissue DCs (CD11b^+^ and CD103^+^), Fc receptors were first blocked using Fc block (BD Biosciences). Next, FITC-labeled CD45^+^ cells from the vasculature were removed using anti-FITC microbeads (Miltenyi Biotec). Following the removal of these cells, remaining cells were first selected (positively or negatively) by incubation with anti-CD11c microbeads (Miltenyi Biotec). Cells positively selected with CD11c were saved for further DC separations, while cells negatively selected were next positively selected using anti-F4/80 microbeads (Miltenyi Biotec) to select for macrophage subsets. The macrophage populations were next incubated with anti-MHC II microbeads (Miltenyi Biotec) – those positively selected are interstitial macrophages, while those negatively selected were subjected to further incubation with biotinylated anti-Ly6c antibody (Invitrogen, Waltham, Massachusetts) followed by selection with anti-biotin microbeads (Miltenyi Biotec) to distinguish Ly6c^+^ monocyte–like macrophages (positively selected) from Ly6c^-^ monocyte-like macrophages (negatively selected). For DC purification, the CD11c^+^ cells were then incubated with anti-F4/80 microbeads (Miltenyi Biotec) to remove any contaminating macrophages. The CD11c^+^ F4/80^-^ cells were then incubated with anti-CD11b microbeads (Miltenyi Biotec). Cells positively selected with CD11b were CD11b^+^ dendritic cells. Negatively selected CD11b cells (CD11c^+^ CD11b^-^) were then incubated with biotinylated anti-CD103 antibody (BD Biosciences) followed by selection with anti-biotin microbeads (Miltenyi), and positively selected cells were CD103^+^ dendritic cells. Cell purity of each population was verified by using Labeling Check Reagent (Miltenyi Biotec), and cell surface markers for each subset and analyzed by flow cytometry using an Acea Novocyte flow cytometer (Acea Biosciences, San Diego, CA). The cell separation scheme is summarized in **Figure 1**. Alveolar macrophages are defined as CD11c^+^, CD11b^-^, F4/80^+^, SiglecF^hi^, CD68^hi^, and interstitial macrophages are F4/80^+^, CD11b^+^, Cd11c^lo^, Gr1^-^, and MHC II^+^. Monocyte-like Ly6c^-^ macrophages are F4/80^+^, CD11b^+^, Cd11c^-^, Gr1^lo^, CD14^+^, MHC II^-^, and Ly6c^-^, while the monocyte-like Ly6c^+^ macrophages have the same markers but are Ly6c^+^ (43, 45, 55, 56). CD11b^+^ DCs are characterized by their high expression of CD11c, MHC class II, and absence of CD103. pDCs are characterized by expression of CD11c, B220 and PDCA-1. Finally, CD103^+^ DCs are characterized by their expression of CD103, CD11c, MHC class II and their lack of CD11b (80).

### Flow Cytometry

Following cell separation, murine macrophage and DC subsets were resuspended in FACS buffer (PBS supplemented with 2% FBS) (100ul/well) and incubated with CD16/CD32 (Mouse BD Fc Block) (BD Biosciences) to block non-specific binding. Then, samples were stained with labeling check reagent (Miltenyi Biotec), CD45-APC/APC-Vio770 (Biolegend), CD11c-PE-Cy7 (BD Biosciences), Siglec F-PerCP-efluor (BD Biosciences), CD11b-FITC (BD Biosciences), F4/80-PE (Miltenyi Biotec), Ly6c-APC (BioLegend), MHC II-Superbright 645 (Invitrogen), CD103-PerCP-efluor (BD Biosciences) CD24-APC (BioLegend) a viability dye (LIVE/DEAD Fixable yellow, Invitrogen). Following this, cells were incubated with antibodies for 30 minutes at 4°C then washed three times with FACS buffer and fixed with 2% formaldehyde. Samples were analyzed on an Acea Novocyte 3000 flow cytometer (Agilent Technologies, Santa Clara, CA), and data were analyzed using NovoExpress software (Agilent Technologies).

### Cryptococcal Inhibition Assay

Each subset of pulmonary macrophages or DCs was purified (as described above), and 2x10^5^ cells/ml of each subset were incubated with 1µg/ml anti-GXM antibody (kind gift from Tom Kozel, University of Nevada Reno) and 1x10^4^ cells/ml of *C. neoformans* strain H99 (20:1 ratio) in 100 µl in triplicate wells for 24 hours at 37°C, 5% CO_2_, as previously described (18, 60, 81, 82). After 24 hours, the samples were centrifuged, and supernatants were removed. Macrophages were lysed for 15 minutes at room temperature using 100 µl sterile water. Samples were then serially diluted and plated on YPD agar. Plates were incubated for 48 hours at 30°C, and cryptococcal CFU were quantified.

### Cryptococcal Association Assay

Each subset of pulmonary macrophages or DCs were separated (as described above), and 2x10^5^ cells/ml of each phagocyte subset were incubated with 1µg/ml anti-GXM antibody (Tom Kozel, University of Nevada Reno) and 1x10^4^ cells/ml of *C. neoformans* strain KN99mCherry (20:1 ratio) in 100 µl in triplicate wells for 2 hours at 37°C, 5% CO_2_. After 2 hours, the samples were stained for cell surface markers (as described in Flow Cytometry methods) and were read on an Acea Novocyte 3000 flow cytometer (Agilent Technologies). We defined cells that “associated” with *C. neoformans* as those double-staining for mcherry, which could indicate either attachment and/or internalization of the fluorescent *C. neoformans*. The percent association of each subset with fluorescent *C. neoformans* was calculated using a Novoexpress software (Agilent Technologies).

### Imagestream Imaging Flow Cytometry

Murine phagocyte subsets were isolated as described above and 2x10^5^ cells/ml of each phagocyte subset were incubated with 1µg/ml anti-GXM antibody (Tom Kozel, University of Nevada Reno) and 2x10^5^ cells/ml *C. neoformans* strain Kn99mCherry (1:1 ratio) in 100 µl in triplicate wells for 2 hours at 37°C, 5% CO_2_. This ratio was chosen in order to maximize visualization of internalized fungal cells by imaging flow cytometry. Cells were stained with the following fluorophores: CD45-FITC/PerCP (BD Biosciences), MHC II-Alexa Fluor 488 (BD Biosciences), F4/80-PE (Invitrogen), CD103-PE (Invitrogen), CD11b-PE (Invitrogen), Siglec F-PE (BD Biosciences), Ly6c-PerCP-Cy5.5 (Novus Biologicals), CD11c-PerCP-Cy5.5(BD Biosciences) for 30 min at 4°C. Cells were washed three times with FACS buffer, followed by fixation with 1% ultrapure paraformaldehyde diluted with FACS buffer. Cells were analyzed in the in the Flow and Image Cytometry Laboratory at The University of Oklahoma Health Sciences Center (OUHSC, Oklahoma City, OK) using the Amnis Imagestream Mark II (Luminex, Austin, TX). Using the Amnis IDEAS 6.2 software (Luminex), the internalization masking feature was used and cells were selected based upon the internalization of KN99mCherry. Of the cells selected, 100 cells of each subset from each experiment were quantified and analyzed for cryptococcal intracellular morphologies consisting of condensed (c-shaped), live, budding, and debris, and cells containing each morphotype were quantified. We considered round and budding cells to be “live” while debris, c-shaped, and condensed are “dead” (81). After quantification, percent killing of all subsets was calculated by counting total cells out of 100 that contained each morphotype multiplied by 100.

### Murine Macrophage and Dendritic Cell Subset Cytokine Analysis

Each subset of pulmonary macrophages or DCs was separated (as described above), and 2x10^5^ cells/ml of each phagocyte subset were incubated with 1µg/ml anti-GXM antibody and 1x10^4^ cells/ml *C. neoformans* strain H99 (20:1 ratio) in 100 µl in triplicate wells for 2 hours at 37°C, 5% CO_2_. This time point was chosen to examine early cytokine production following phagocyte-cryptococcal interaction. Following centrifugation, cell supernatants were collected, and protease inhibitor (Thermo Scientific) was added to supernatants. Treated supernatants were placed in tubes and stored at -80°C until analysis. Supernatants were assayed for mouse cytokines/chemokines according to manufacturer’s instructions using the Bio-Plex Pro Mouse Cytokine 23-plex (BioRad, Hercules, CA) and samples were read on a BioRad BioPlex^®^-200 (Bio-Rad). Data were analyzed using GraphPad Prism v5.0 (GraphPad Software, San Diego, CA).

### RNA Sequencing of Phagocyte Subsets Following Interaction With *C. neoformans*

Following incubation of each macrophage or DC subset with *C. neoformans* strain H99 at a ratio of 20:1 and 1µg/ml anti-GXM antibody at 37°C, 5% CO_2_ for 2h, cells were collected and stored in Trizol (Invitrogen) at -20°C until analysis. This time point was chosen to examine early gene transcription changes following incubation of phagocytes with *C. neoformans*. RNA purification was conducted by Novogene (Novogene Corp, Sacramento, CA). Next, murine RNA-sequencing was conducted using SMARTer Stranded V2 library prep and samples were sequenced on the Illumina Platform (PE150 Q30≥80%) (Novogene Corp). Gene expression was compared between each macrophage and DC subset alone and the same subset when incubated with *C. neoformans* strain H99. Significant differences within gene expression were calculated using readcount adjusted by trimmed mean of M-values (TMM), then differential significant analysis was performed using the edgeR package (Bioconductor, Fred Hutchinson Cancer Research Center, Seattle, WA) within R Studio (Delaware Public Benefit Corporation (PBC), Boston, MA), with the significant criterion being both qvalue < 0.005 and |log2(FoldChange)| > 1. Genes with significantly different expression values were grouped into signaling pathways using KEGG Pathway Analysis (Kanehisa Laboratories, Kyoto, Japan) and Ingenuity Pathway Analysis (IPA) software (Qiagen).

### Statistical Analysis

GraphPad Prism v5.0 (GraphPad Software, San Diego, CA) was used to detect significant differences between CFU of H99 alone compared to each test group within the cryptococcal inhibition assay by an unpaired two-tailed T test with a 95% confidence value with a p-value consisting of *p* < 0.05. Differences in fungal/phagocyte association and cytokine production were analyzed using one-way ANOVA with Tukey’s post-test, with significant differences defined as *p <*0.05. IDEAS 6.2 software (Luminex Corporation), was used to analyze and quantify intracellular cryptococcal morphologies out of a total of 100 phagocytes per subset. Significant differences in intracellular cryptococcal morphology was calculated with GraphPad Prism using an unpaired two-tailed T test with a 95% confidence value with a p-value consisting of *p* < 0.05. Significant differences within gene expression were calculated using sequencing readcount adjusted by the trimmed mean of M-values performed using the edgeR package (Bioconductor). Gene significance criterion was determined using both q-value <0.005 and |log2(FoldChange)| > 1. Genes with significantly different expression values were grouped into signaling pathways using KEGG Pathway Analysis.

## Results

### Murine Pulmonary Phagocyte Subsets Exhibit Varying Fungicidal Activity

**Figure 2:**
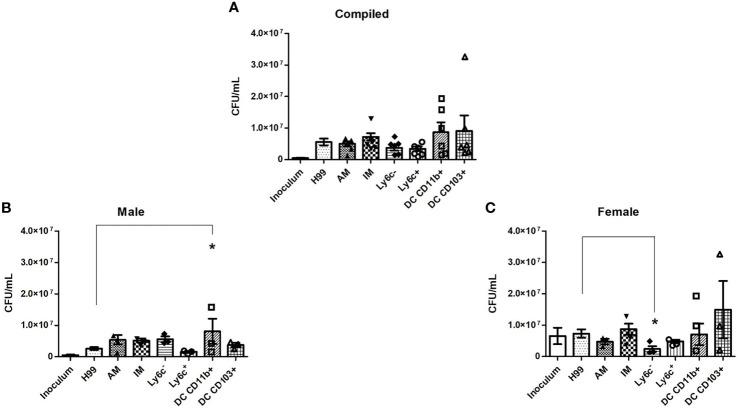
Murine pulmonary phagocyte subsets exhibit differing anti-cryptococcal capabilities dependent upon sex. Pulmonary phagocyte subsets were purified from both male and female BALB/c mice and incubated with *C. neoformans* strain H99 for 24h at 37°C, 5% CO_2_ at a ratio of 20:1. Macrophages or DCs were lysed with sterile water, then contents were diluted and plated on YPD agar. Plates were incubated for 48h at 30°C and CFUs were counted. **(A)** CFU counts from combined experiments from male and female phagocyte populations following *C. neoformans* strain H99 incubation alone (H99), with alveolar macrophages (AM), with interstitial macrophages (IM), with Ly6c^-^ monocyte-like macrophages (Ly6c^-^), Ly6c^+^ monocyte-like macrophages (Ly6c^+^), CD11b^+^ DCs and CD103^+^ DCs. Inoculum indicates initial *C. neoformans* strain H99 concentration prior to incubation. **(B)** CFU counts from male phagocyte populations following incubation with *C. neoformans* strain H99. **(C)** CFU counts from female phagocyte populations following incubation with *C. neoformans* strain H99. The data are means ± standard errors of the means (SEM) for three independent experiments (n=3) each of both 20 male and 20 female mice (cells from 20 mice pooled for each experiment). Significant differences (*) shown represent *p* < 0.05.

To evaluate the fungicidal activity of phagocyte subsets within the murine pulmonary cavity, subsets of pulmonary macrophages (alveolar, interstitial, Ly6c^-^ monocyte-like and Ly6c^+^ monocyte-like) and conventional DCs (CD11b^+^ and CD103^+^) were separated (**Figure 1**) and used in anti-cryptococcal assays. The fungicidal activity of these populations was assessed as shown in **Figure 2**. Statistical analysis revealed that there was no significant difference in antifungal activity following incubation of *C. neoformans* with any phagocyte population compared to *C. neoformans* strain H99 alone (**Figure 2A**). In order to eliminate sex as a biological variable, and because our experiments were performed in both male and female mice, we were able to stratify the data by sex. Once we analyzed the sex-stratified data, the male CD11b^+^ DC population led to significant increase of cryptococcal growth (*p*=0.03) (**Figure 2B**), while incubation with the female DC populations did not significantly affect cryptococcal growth as compared to *C. neoformans* strain H99 alone (**Figure 2C**). In addition, incubation of *C. neoformans* with the female Ly6c^-^ monocyte-like macrophages significantly inhibited cryptococcal growth (*p*=0.03) compared to *C. neoformans* grown in media alone (**Figure 2C**). These results demonstrate that murine pulmonary phagocyte subsets interact differently with *C. neoformans*, and the sex of the host is a factor.

### Murine Pulmonary Phagocyte Subsets Interact With *C. neoformans*

**Figure 3:**
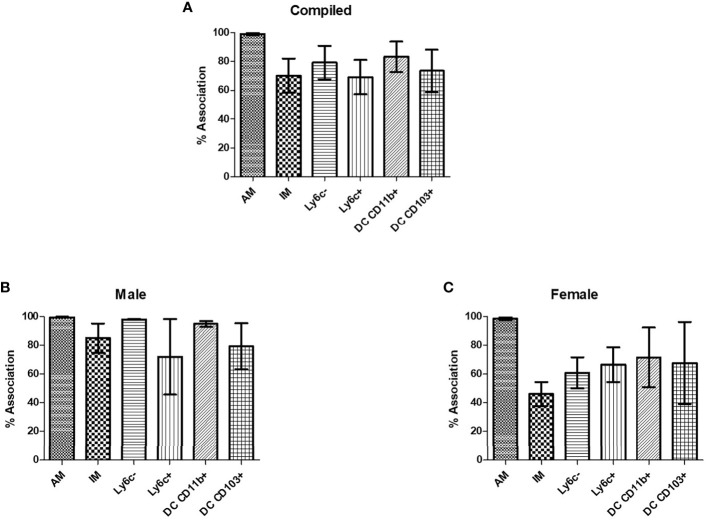
Murine pulmonary phagocyte subsets interact with *C. neoformans ex vivo*. Pulmonary phagocyte subsets were purified from both male and female BALB/c mice (20 mice pooled per experiment) and incubated with mcherry expressing *C. neoformans* (Kn99mCH) for 2hrs at 37°C, 5% CO_2_ at a ratio of 1:1. Following incubation, cells were stained for surface markers and analyzed for cryptococcal association *via* flow cytometry. Association is defined as cryptococcal cells internalized and/or attached to the phagocytes. **(A)** Percent association of *C. neoformans* strain H99 with each phagocyte subset from combined experiments from both male and female mice. **(B)** *C. neoformans* strain H99 association percentages for male phagocyte populations. **(C)** *C. neoformans* strain H99 association percentages for female phagocyte populations. Alveolar macrophage (AM), interstitial macrophage (IM), Ly6c^-^ monocyte-like macrophages (Ly6c^-^), Ly6c^+^ monocyte-like macrophages (Ly6c^+^), CD11b^+^ DCs, and CD103^+^ DCs. The data are means ± standard errors of the means (SEM) for three independent experiments (n=3) each from both male and female mice.

In order to verify that *C. neoformans* interacted with murine pulmonary phagocyte subsets equally (and that any differences in antifungal activity were not attributable to differences in interaction), both male and female pulmonary macrophage and DC subsets were incubated with an mcherry expressing *C. neoformans* strain (KN99mCH) for 2 hours at 37°C, 5% CO_2_ at a ratio of 20:1. Following incubation, cells were stained with antibodies to cell surface markers and examined by flow cytometry. When analyzing macrophage and DC subsets and *C. neoformans* association (internalization and/or attachment), data showed that all subsets interacted with *C. neoformans*, and there were no significant differences in interaction between the populations (**Figure 3A**), even when sexes were stratified (**Figures 3B, C**). These results show that all pulmonary phagocyte subsets have the ability to interact with *C. neoformans ex vivo*, suggesting that differences in antifungal activity (**Figure 2**) are not due to the inability of specific subsets to interact with the fungus.

### Internalized *C. neoformans* Display Varying Cryptococcal Morphologies When Interacting With Pulmonary Phagocyte Subsets

**Figure 4:**
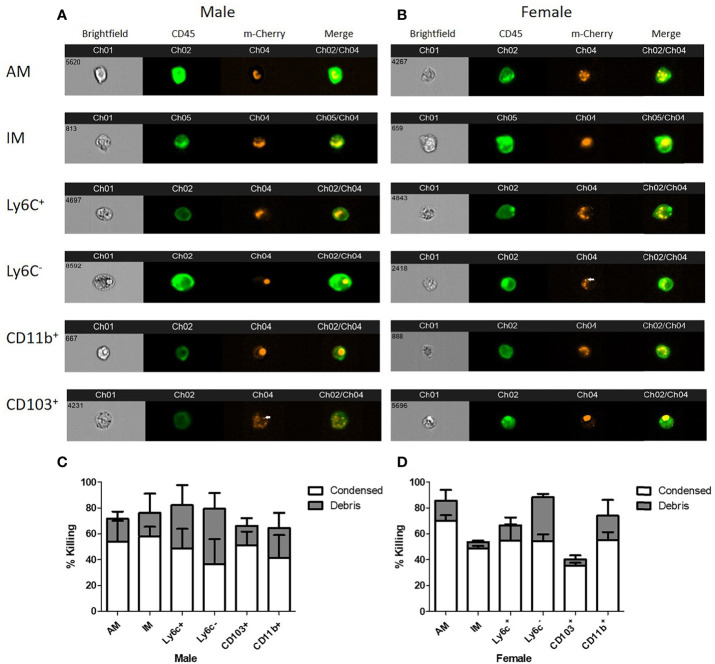
Murine pulmonary phagocyte subsets display varying cryptococcal morphologies after *C. neoformans* internalization. Using female or male BALB/c mice (pooled tissues from 20 mice each), phagocyte subsets were purified into individual subsets and incubated with mcherry expressing *C. neoformans* (KN99Mch) for 2h at a 1:1 ratio. Following incubation, cells were stained for CD45 and imaged at 40x magnification using the ImageStreamX-Imaging Flow Cytometer-MKII (Luminex). **(A, B)**. Cryptococcal morphologies within each pulmonary phagocyte subset from male **(A)** and female **(B)** mice. Arrows point to condensed cryptococci. **(C, D)**. Quantitative data of cryptoccocal morphology using IDEAS Software 6.2 are shown for the compiled experiments in male **(C)** and female **(D)** mice. Alveolar macrophage (AM), interstitial macrophage (IM), Ly6c^-^ monocyte-like macrophages (Ly6c^-^), Ly6c^+^ monocyte-like macrophages (Ly6c^+^), CD11b^+^ DCs (CD11b), and CD103^+^ DCs (CD103). The data are means ± standard errors of the means (SEM) for three independent experiments (n=3) in male and female mice.

In order to determine the intracellular morphology of *C. neoformans* following the pulmonary phagocyte subset interaction, imaging flow cytometry was used. Both male and female pulmonary phagocyte subsets were purified (as described in **Figure 1**) and incubated with an mcherry expressing *C. neoformans* strain (KN99mCH) for 2 hours at 37°C, 5% CO_2_ at ratio of 1:1 – this ratio was used to maximize visualizing intracellular organisms in the phagocyte populations. Following incubation, cells stained for the CD45^+^ leukocyte marker and cryptococcal morphologies were examined at a 40x magnification by the ImageStreamX-Imaging Flow Cytometer-MKII (Luminex). **Figures 4A, B** show examples of intracellular cryptococcal morphologies of cryptococcal cells within both male and female phagocyte populations, respectively. We observed distinct cryptococcal morphologies including circular, budding, crescent-shaped (c-shaped), condensed, and debris. A circular or budding cryptococcal morphology is indicative of living and/or replicating cryptococcal yeast cells. The crescent-shaped morphology, condensed morphology and intracellular debris indicates dead and/or dying cryptococcal yeast cells (81). Structural changes in the cell membrane of live *C. neoformans* from circular to a crescent shaped morphology due to the rupturing of the cell wall is a possible indication of cryptococcal death (81). Data revealed that all phagocyte subsets had a variety of intracellular cryptococcal morphologies following a 2h incubation with the mcherry-expressing *C. neoformans* strain (**Figures 4A, B**). Using IDEAS 6.2 software (Luminex), images were quantified as described in Materials and Methods. Cells with internalized cryptococci were counted and the number of cells with each morphology was recorded as a percentage of the total (**Figures 4C, D**).

### Murine Phagocyte Fungicidal Activity Is Not Dependent Upon Phagocyte Polarization

**Figure 5:**
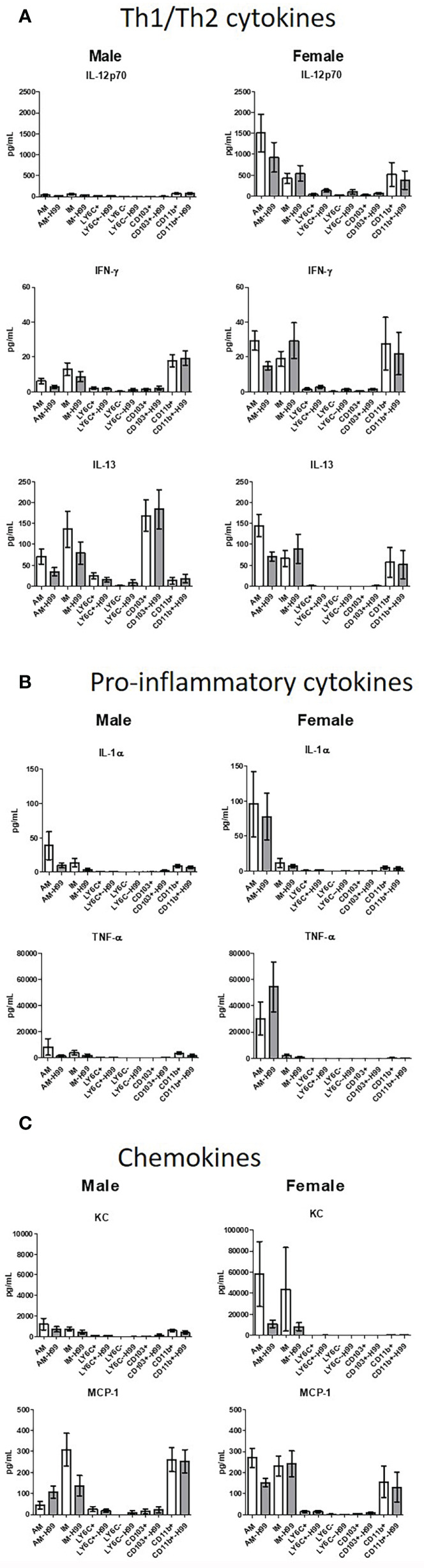
Pulmonary phagocyte cytokine production following incubation with *C. neoformans*. Murine pulmonary macrophages and DCs were harvested from pooled tissues from 20 mice/experiment and purified using magnetic separation. Cell subsets were incubated at a 20:1 ratio with *C. neoformans* for 2h at 37°C, 5% CO_2_ then supernatants were collected. Samples were treated with protease inhibitor and frozen at -80°C until analysis. Samples were analyzed for cytokines/chemokines by Bioplex (BioRad). **(A)** Th1/Th2 cytokine secretion in pg/ml from both male and female phagocyte subsets alone (clear bars) and when incubated with *C. neoformans* strain H99 (gray bars). **(B)** Pro-inflammatory cytokine secretion in pg/ml from both male and female phagocyte subsets alone (clear bars) and when incubated with *C. neoformans* strain H99 (gray bars). **(C)** Chemokine secretion from both male and female phagocyte subsets alone (clear bars) and when incubated with *C. neoformans* strain H99 (gray bars). Data are shown as pg/ml ± standard errors of the means (SEM) from 3 independent experiments each from male mice and female mice (n=3 experiments in each male and female mice). AM = alveolar macrophages, M = interstitial macrophages, LY6N = Ly6c negative monocyte-like macrophages, LY6P= Ly6c positive monocyte-like macrophages, CD11b = CD11b^+^ dendritic cells, CD103 = CD103^+^ dendritic cells.

In order to determine if exposure to *C. neoformans* induced cytokine production and/or polarization of macrophage or DC subsets at an early time point following cryptococcal exposure, cytokine production was examined. Following a 2-hour incubation with *C. neoformans*, cytokines present in the supernatants were assayed using the Bio-Plex Pro Mouse Cytokine 23-plex (BioRad). Th1/Th2 cytokines, pro-inflammatory cytokines, and chemokine levels from the supernatant of each subset were quantified as shown in **Figure 5**. Cytokines were measured from cells cultured *ex vivo* without the influence of the lung microenvironment, so the cytokines measured in these studies were produced by each subset as a response to *C. neoformans* alone. Concentrations were measured from both male and female pulmonary phagocyte subset populations alone and when incubated with *C. neoformans* strain H99. When analyzing Th1/Th2 associated cytokine levels (IL-12p70, IFN-*γ*, IL-13), neither male nor female phagocyte subsets displayed any significant differences in cytokine production when comparing the phagocyte population alone and when incubated with *C. neoformans* strain H99 as shown in **Figure 5A**. Next, when analyzing pro-inflammatory cytokines (IL-1α, TNF-α) there were no significant differences in cytokine production from male or female phagocyte subsets incubated alone compared to the same subset incubated with *C. neoformans* strain H99 (**Figure 5B**). Lastly, when analyzing chemokines (KC, MCP-1) neither male nor female phagocyte subset populations displayed any significant differences in chemokine secretion when comparing phagocyte populations alone and when incubated with *C. neoformans* strain H99 as shown in **Figure 5C**. We noticed that cytokine/chemokine production was higher in cells from female mice. Upon analyses from the same subset from male *vs* female mice, we found that TNF-α, KC, and MCP-1 were produced in significantly higher amounts in female alveolar macrophages (AM) *vs* male AMs, but after incubation with *C. neoformans*, these differences were no longer present. However, IL-12p70 was produced in significantly higher amounds from female AM *vs* male AM, and this continued even after incubation with *C. neoformans*. IL-13 was also produced at significantly higher levels by female CD103^+^ DCs *vs* male CD103^+^ DCs, and this also continued after incubation with *C. neoformans* (**Figure 5**). The results indicate that pulmonary phagocytes from female mice inherently produce higher amounts of some cytokines. In addition, these data show that – in this experimental system - phagocyte polarization is not responsible for the differences in fungicidal activity exhibited by pulmonary macrophage and dendritic cell subsets *ex vivo*.

### RNA Sequencing Analysis of Murine Pulmonary Phagocytes Displayed Differences in Transcriptional Profiles Within Permissive and Non-Permissive Phagocytes

**Figure 6:**
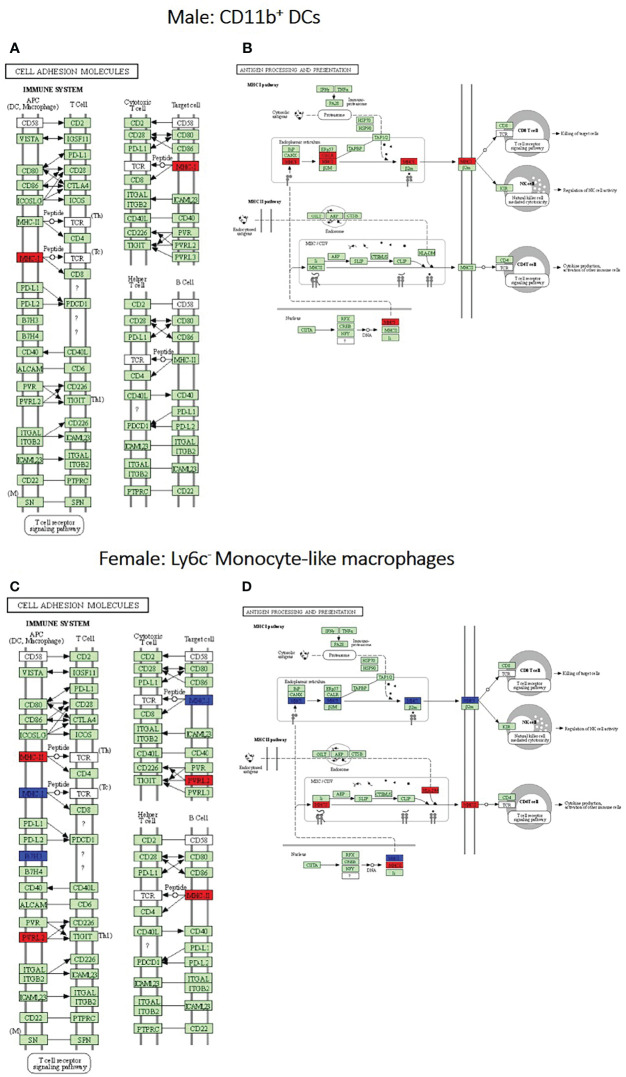
Permissive and non-permissive (antifungal) pulmonary phagocytes exhibit transcriptional differences in immune associated pathways. Using male and female BALB/c mice, phagocyte subsets were purified and incubated with *C. neoformans* strain H99 at 37°C, 5% CO_2_ for 2hrs at a ratio of 20:1. Following incubation, cells were collected and stored in TriZol at -20°C until analysis. RNA purification and analysis was conducted by Novogene using SMARTer Stranded V2 library prep and samples were sequenced using the Illumina Platform. Differential expression analysis was compared between each macrophage and DC subset alone and following incubated with *C. neoformans* strain H99. Significant differences in gene expression were identified and grouped into signaling pathways using KEGG Pathway Analysis. **(A, B)**. Up- and down-regulation of genes associated with male CD11b^+^ dendritic cells (DCs) within the cell adhesion molecules and antigen processing and presentation signaling pathways, respectively. **(C, D)**. Up- and down-regulation of genes associated with female Ly6c^-^ monocyte-like macrophages within the cell adhesion molecules and antigen processing and presentation signaling pathways, respectively. Blue and red represent genes up-regulated or down-regulated, respectively, in CD11b^+^ DCs and Ly6c^-^ monocyte-like macrophages interacting with *C. neoformans* compared to the subset alone. Data were generated from a merged data set (n=2) from 2 independent female and 2 independent male mouse experiments.

**Figure 7:**
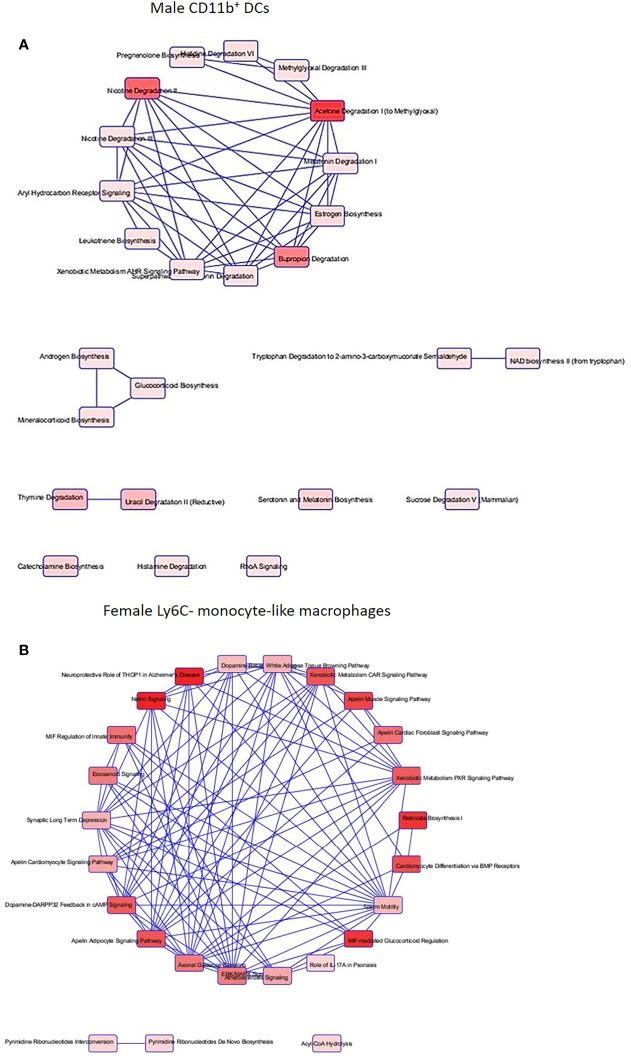
*C. neoformans* leads to a highly differentiated network of associated metabolic pathways within permissive phagocytes and fungicidal (non-permissive) phagocytes. Using male and female BALB/c mice, phagocyte subsets were purified and incubated with *C. neoformans* strain H99 at 37°C, 5% CO_2_ for 2hrs at a ratio of 20:1. Following incubation, cells were collected and stored in TRIzol at -20°C until analysis. RNA purification and analysis was conducted by Novogene using SMARTer Stranded V2 library prep and samples were sequenced using the Illumina Platform. Differential expression analysis was compared between each macrophage and DC subset alone and when incubated with *C. neoformans* strain H99. Significant differences within gene expression were identified and grouped into predicted canonical (active) pathways using Ingenuity Pathway Analysis (IPA) software. **(A)** Associated pathways containing overlapping genes when comparing male CD11b^+^ DCs interacting with *C. neoformans* to CD11b^+^ DCs alone. **(B)** Associated pathways containing overlapping genes when comparing female Ly6c^-^ monocyte-like macrophages interacting with *C. neoformans* to Ly6c^-^ monocyte-like macrophages alone. Connecting lines indicate pathways with shared genes. Boxes shaded from dark red to light red indicates a difference *p* < 0.05 from greatest to least, respectively. Data were generated from 2 independent female and 2 independent male mouse experiments (n=2 male and female experiments).

After identification of phagocyte subsets with different antifungal activity from male and female BALB/c mice (**Figure 2**), we were interested in identifying genes involved in these responses. Following a 2-hour incubation with *C. neoformans*, RNA from both male and female phagocyte subsets was collected, and RNA sequencing was performed. Initial analyses of each subset from each sex with and without *C. neoformans* exposure revealed a range of genes up- and down-regulated (from 39 to 1816) in each subset (**Table 1**). The top 10 genes affected in each phagocyte subset following interaction with *C. neoformans* are listed **Supplemental Table 1**. In order to narrow our search to genes involved specifically in permissive growth *vs* fungicidal activity, gene expression in permissive phagocytes (male CD11b^+^ DCs) *vs* non-permissive, fungicidal phagocytes (female Ly6c- monocyte-like macrophages) were compared to identify differentially regulated pathways. As shown in **Figure 6**, using the KEGG Pathway database, immune-associated response pathways, cell adhesion molecules and antigen processing and presentation, were identified within permissive and non-permissive (antifungal) phagocytes and compared. We chose to focus on the cell types with the most significant differences in activity – the CD11b^+^ DCs from male mice (permissive, see **Figure 2B**) and Ly6c^-^ monocyte-like macrophages from female mice (antifungal/non-permissive, see **Figure 2C**). When examining the up- and down-regulated genes from these subsets, we found that these shared common immune associated signaling pathways that included the cell adhesion molecule pathway and the antigen processing and presentation pathway (**Figure 6**). Within the cell adhesion molecule pathway, gene expression analysis revealed a down-regulation in MHC-1 (red) within the CD11b^+^ DCs from male mice (**Figure 6A**). In the antigen processing and presentation pathway, within CD11b^+^ DCs from male mice, there was a down regulation in both MHC-I (red) and CALR (red) as shown in **Figure 6B**. When analyzing the same pathway within Ly6c^-^ monocyte-like macrophages from female mice, there was an up-regulation in MHC-I (blue) and B7H3 (blue), and down-regulation in both MHC-II (red) and PVRL2 (red) as shown in **Figure 6C**. When analyzing the same pathway within female mice, there was an upregulation in MHC-I (blue) and a down-regulation in both MHC-II (red) and HLA-DM (red) as shown in **Figure 6D**. Notably, within both shared pathways, MHC-I was significantly down-regulated (*p* < 0.05) within the permissive CD11b^+^ DC subsets from male mice and significantly up-regulated (*p* < 0.05) within Ly6c^-^ monocyte-like macrophage subset from female mice. Using Ingenuity Pathway Analysis (IPA) software, top canonical (active) pathways associated with cell metabolism were predicted and their associated genes were identified for both permissive and non-permissive phagocyte subsets as shown in **Tables 2**, **3**. Within permissive phagocytes (male CD11b^+^ DCs), five top canonical (active) metabolic pathways were identified that included Acetone Degradation I (to Methylglyoxcal) (*p*=1.25E^-03^), Nicotine Degradation II (*p*=8.60E^-03^), Bupropion Degradation (*p*=1.21E^-02^), Uracil Degradation II (reductive) (*p*=2.28E^-02^) (**Table 2** and **Figure 7A**), and Thymine Degradation (*p*=2.28E^-02^). Within these 5 metabolic pathways, their genes associated within permissive phagocytes were identified along with their up-regulation or down-regulation (+/-) (**Table 2**). Within non-permissive (antifungal) phagocytes (female Ly6c^-^ monocyte-like macrophages), the top 5 canonical (active) metabolic pathways that were identified included Netrin Signaling (*p*=3.3E^-03^), Neuroprotective Role of THOP1 in Alzheimer’s Disease (*p*=4.31E^-03^), MIF-mediated Glycocorticoid Regulation (*p*=6.50E^-03^), Retinoate Biosynthesis I (*p*=6.50E^-03^), and the Apelin Muscle Signaling Pathway (*p*=8.02E^-03^) as shown in **Table 3** and **Figure 7B**. The specific genes within each of these pathways associated with non-permissive phagocytes were also identified along with their up-regulation or down-regulation (+/) (**Table 3**). Using the Ingenuity Pathway Analysis platform, a broader analysis was completed in order to identify over lapping canonical pathways based upon shared significantly differentiated genes within permissive and non-permissive (antifungal) phagocyte subsets (**Figures 7A, B**). Within permissive phagocytes, 25 active metabolic pathways were identified (**Figure 7A**). Lines connecting pathways represent metabolic pathways with shared genes. **Figure 7A** shows a network of associated metabolic pathways, within permissive phagocytes, based upon shared genes. The most active pathways are ranked from p-values smallest to largest indicated by dark red to light red shading. Within this network the most active pathways, shown in dark red, are Acetone Degradation I (to Methylglyoxal), Nicotine Degradation II, and Bupropion Degradation including lines connecting each pathway to related pathways that have shared genes. Similarly, within non-permissive (antifungal) phagocytes, 25 active metabolic pathways were identified as shown in **Figure 7B**. Lines connecting pathways represent metabolic pathways with shared genes. **Figure 7B** shows a network of associated metabolic pathways within non-permissive phagocytes based upon shared genes. The most active pathways are ranked from p-values smallest to largest indicated by dark red to light red shading, respectively. Within this network the most active pathways, shown in dark red, are Netrin Signaling, Neuroprotective Role of THOP1 in Alzheimer’s Disease, and MIF-mediated Glucocorticoid Regulation including lines connecting each pathway to related pathways that have shared genes (**Figure 7**). The results indicate the *C. neoformans* causes distinct transcriptional changes within permissive and non-permissive (antifungal) phagocytes.

**Table 1:** Genes Up- and Down-regulated After Incubation with *C. neoformans*.

Cell type	Sex	# genes up-regulated	# genes down-regulated
AM	Male	39	46
AM	Female	45	114
IM	Male	78	104
IM	Female	68	44
Ly6c^-^	Male	1262	1332
Ly6c^-^	Female	773	142
Ly6c^+^	Male	542	272
Ly6c^+^	Female	139	142
CD11b^+^	Male	208	187
CD11b^+^	Female	173	174
CD103^+^	Male	1003	1816
CD103^+^	Female	55	50

Total number of genes significantly up- or down-regulated (p <0.05). AM, alveolar macrophages; IM, interstitial macrophages; Ly6c^-^, Ly6c^-^ monocyte-like macrophages; Ly6c^+^, Ly6c^+^ monocyte-like macrophages; CD11b^+^, CD11b^+^ dendritic cells; CD103^+^, CD103^+^ dendritic cells.

**Table 2:** *C. neoformans* leads to the activation of highly specific metabolic pathways in male CD11b^+^ permissive DCs.

Pathway Name	*p*-value	Genes
Acetone Degradation I (to Methylglyoxal)	1.25E10^-03^	**CYP1B1**: Cytochrome P450 family 1 subfamily B member 1 (+) **CYP2U1**: Cytochrome P450 family 2 subfamily U member 1 (+) **CYP4A22**: Cytochrome P450 family 4 subfamily A member 22 (+)
Nicotine Degradation II	8.6E10^-03^	**CYP1B1**: Cytochrome P450 family 1 subfamily B member 1 (+) **CYP2U1**: Cytochrome P450 family 2 subfamily U member 1 (+) **Fmo9**: Flavin containing monooxygenase 9 (+)
Buproprion Degradation	1.21E10^-03^	**CYP1B1**: Cytochrome P450 family 1 subfamily B member 1 (+) **CYP2U1**: Cytochrome P450 family 2 subfamily U member 1 (+)
Uracil Degradation II (reductive)	2.28E10^-03^	**DPYS**: dihydropyrimidase (+)
Thymine Degradation	2.28E10^-03^	**DPYS**: dihydropyrimidase (+)

Top 5 most active pathways order p-value least to greatest. Genes are followed by upregulation (+) or downregulation (–).

**Table 3:** *C. neoformans* leads to the activation of highly specific metabolic pathways in female Ly6c^-^ antifungal monocyte-like macrophages.

Pathway Name	p-value	Genes
Netrin Signaling	3.13E10^-03^	**ABLIM3**: actin binding LIM protein family member 3 (+) **CACNB4**: calcium voltage gated channel auxiliary subunit beta 4 (+) **CACNG8**: calcium voltage gated channel auxiliary subunit gamma 8 (+) **PPP3R2**: protein phosphatase 4 regulatory subunit beta B, beta (+) **PRKAR1B**: Protein kinase cAMP-dependent type 2 regulatory subunit beta (+) **RAC3**: Rac family small GTPase3 (–)
Neuroprotective Role of THOP1 in Alzheimer’s disease	4.31E10^-03^	**C1R**: complement C1r (+) **CTRL**: chymotrypsin like (–) **GZMM**: granzyme M (–) **KLK6**: kallikrein related peptidase 6 (–) **PRKAR1B**: protein kinase cAMP-dependent type 1 regulatory subunit beta (+) **PRSS50**: serine protease 50 (–) **PRSS58**: serine protease 58 (–) **TMPRSS11**: transmembrane serine protease 11A (–)
MIF-mediated Glucocorticoid Regulation	6.50E10^-03^	**PLA2G2E**: phospholipase A2 group IIE (+) **PLA2G2F**: phospholipase A2 group IIF (+) **PLATG4F**: phospholipase A2 group IVF (+) **PTGS2**: prostaglandin- endoperoxide synthase 2 (–)
Retinoate Biosynthesis I	6.5E10^-03^	**ADH4**: alcohol dehydrogenase 4 (class II), pi polypeptide (+) **BMP2**: bone morphogenetic protein 2 (–) **RDH16**: retinol dehydrogenase 16 (–)
Apelin Muscle Signaling Pathway	8.02E10^-03^	**APLN**: Apelin (+) **APLNR**: Apelin receptor (–) **PPARGC1A**: PPARG coactivator 1 alpha (+)

Top 5 most active pathways order p-value least to greatest. Genes are followed by upregulation (+) or downregulation (–).

## Discussion

Although the induction of HAART therapy has resulted in declining HIV/AIDS related mortality, *C. neoformans* still remains the leading cause of fungal related death in these patients (83). The identification of specific mechanisms that mediate intracellular *C. neoformans* survival and replication can aid in the development of novel therapies for the treatment of cryptococcosis. Previous studies have provided an investigation into the intracellular nature of *C. neoformans* and phagocytic cells using peripheral blood mononuclear cells (PBMCs), or macrophage cell lines (4, 84). In addition, more recent fate-mapping studies have identified macrophage and DC subsets and have defined origins of both human and murine phagocytes (56, 85, 86) but few have analyzed how these phagocyte subsets initially interact with *C. neoformans *[reviewed in (60)]. The bacterial pathogen, *Mycobacterium tuberculosis* (Mtb) has similar lung pathology, can survive intracellularly in macrophages, and has similar protective immune responses to *C. neoformans* [reviewed in (87, 88)]. Studies have shown that alveolar macrophages are more permissive to intracellular bacterial growth of Mtb as opposed to interstitial macrophages, resulting in differences in transcriptional responses upon the interaction with Mtb. Permissive alveolar macrophages show an upregulation of genes involved in iron sequestration and fatty acid use. Interstitial macrophages exhibit an upregulation of nitric oxide and genes involved in limiting the availability of iron to the phagosome (69, 70, 89).

In our study, we first examined the fungicidal activity of each murine pulmonary phagocyte subset. We demonstrated that pulmonary phagocyte subsets respond differently upon interaction with *C. neoformans*, and this was dependent on the sex of the host. This has correlations to human patients, as males are more affected by *C. neformans* related infection more frequently and more severely than females [as reviewed in (90)]. Once our data were stratified by sex, results showed a significant decrease in the amount of cryptococcal growth when *C. neoformans* strain H99 was incubated with female Ly6c^-^ monocyte-like macrophages as compared to the H99 control, but this was not seen within the male phagocyte population. In a reversible hepatic fibrosis murine model, it was shown that Ly6c^-^ monocyte like-macrophages played a crucial role in resolving inflammation and showed an enrichment for pathways related to lysosomes, endocytosis and antigen presentation which are implicated in phagocytosis (91, 92). Female Ly6c^-^ monocyte-like macrophages could exhibit a similar role in resolving pulmonary inflammation and controlling cryptococcal growth during a *C. neoformans* infection. Based on these results several studies have been initiated in our lab to confirm these findings and to gain more insight into the *C. neoformans* and pulmonary phagocyte interaction.

In our studies, an association analysis (which included fungi that were attached and/or internalized) was performed with the use of flow cytometry in order to verify that each pulmonary phagocyte subset was able to interact with *C. neoformans*, and that our antifungal activity was not biased by the lack of interaction by individual subsets. We confirmed that all murine pulmonary phagocyte subsets interacted with *C. neoformans ex vivo* to a similar degree. Therefore, our differences in antifungal activity were not attributable to differences in degree of phagocyte-cryptococcal interactions.

To observe early pulmonary phagocyte-cryptococcal interaction, we conducted imaging flow cytometry experiments. This technology allowed us to quantify intracellular morphologies within each subset. These studies, conducted at 2h, showed that most phagocytic subsets had antifungal activity. However, by 24h these phenotypes had changed (**Figure 2**). These data correlate to other published studies showing that *C. neoformans* can manipulate host cells using a variety of virulence factors and other mechanisms (25, 28). It is quite probable that most phagocyte subsets have initial antifungal activity until the fungal cells are able to begin manipulating the interaction, and the outcome ultimately depends on how the phagocyte responds to this manipulation. In addition, we had to use a 1:1 ratio for these experiments in order to identify enough phagocytes with internalized cryptococci for downstream analyses, which we acknowledge may skew the data. However, since we observed more cells that appear to be dying at this ratio, we do not think the increase in number of cryptococcal cells made an impact on the early ability of the cells to have initial antifungal activity. Interestingly, our gene expression data show that the changes in gene expression are already apparent within 2 hours, indicating that the cells were already beginning to have disparate responses, even though they each appeared to initially have antifungal activity.

Previous literature has shown that phagocyte polarization (specifically macrophage polarization) is critical for controlling cryptococcal infection. Protective responses in a murine vaccination model are highly dependent upon cell mediated Th1-type immune responses resulting in M1 macrophage polarization (12, 93, 94). Permissive growth of *C. neoformans* is related to the induction of a Th2-type response, leading to the induction of M2 macrophage polarization and disease exacerbation (15, 74, 95). In our study, we measured cytokines in the supernatant of each pulmonary phagocyte subset with/without *C. neoformans* in order to identify cytokine production and possible phagocyte polarization. In cells from both male and female mice, we did not observe any significant decreases in the amount of secreted Th1/Th2 associated cytokines IL-12p40, IL-13, and IFN-γ or chemokines MIP-1a and MCP-1 when comparing phagocyte subsets alone and when incubated with *C. neoformans* strain H99. Similarly, when observing pro-inflammatory cytokine secretion of IL-1a and TNF-a there were no significant differences seen in cytokine secretion by any phagocyte population alone compared to those incubated with *C. neoformans* strain H99. Interestingly, some cytokines were produced in higher amounts by cells from female mice compared to male mice (AM – TNF-α, IL-12p70, KC, MCP-1; CD103^+^ DCs – IL-13), but only IL=13 and IL-12p70 continued to be significantly increase in these cells following incubation with *C. neoformans*. Although we did observe some minor changes in overall cytokine production by pulmonary phagocytes, none correlated to polarization of macrophages or DCs. However, it should be noted that these cells were only examined *ex vivo*, without the influence of other immune cells or the lung microenvironment. Therefore, we concluded that in this experimental system, interaction of phagocyte subsets with *C. neoformans ex vivo* does not lead to polarization of these cells.

In order to analyze differences within the transcriptional profiles of permissive and non-permissive phagocyte subsets, we conducted an RNA sequencing analysis comparing each phagocyte subset upon the interaction with *C. neoformans* to the subset alone. Although it is possible that our positive-selection separations could affect gene expression, each individual subset of cells was treated in the same manner until interaction with *C. neoformans*. Differential gene expression analyses were only conducted between the cell type without *C. neoformans* exposure compared to the same cell type exposed to *C. neoformans* incubated under the same conditions. Therefore, any changes observed in gene expression were solely due to *C. neoformans* exposure. Within permissive and non-permissive phagocytes, we discovered a large network of differentiated metabolic pathways sharing common genes that were significantly up-regulated and down-regulated. Further studies are needed to provide a more in-depth analysis into these pathways in order to determine their involvement in regards to fungicidal activity within permissive and non-permissive (antifungal) phagocytes. In addition, within the permissive male phagocyte subset CD11b^+^ DCs we observed a significant down regulation of MHC-1 associated with antigen processing and presentation and cell adhesion molecules. Interestingly, this expression was opposite in the non-permissive (antifungal) female Ly6c^-^ monocyte-like macrophages which demonstrated a significant upregulation of MHC-1. The major histocompatibility (MHC) class 1 antigen presentation pathway is most commonly associated with initiating adaptive responses to virally infected cells [reviewed in (96)]. MHC Class I molecules are present on the cell surface of all nucleated cells [reviewed in (97)] and function to present foreign and antigenic internalized peptides primarily to CD8^+^ T lymphocytes. However, in the case of extracellular pathogens, some phagocytes are capable of cross-presenting antigens derived from the phagolysosome and shuttling them into the MHC class I processing pathway (98–101). Evidence that permissive phagocytes show a down-regulation in MHC class I serves as an indicator that *C. neoformans* leads to transcriptional changes which may contribute to a deficiency in vital immune cell machinery that aids to control and eliminate the fungal pathogen. Our pathway analysis revealed that the top pathways activated related to various metabolic functioning within permissive and non-permissive (antifungal) phagocytes. The results revealed multiple distinct metabolic pathways linked by common gene similarities. We concluded that *C. neoformans* leads to differential gene expression affecting multiple metabolic pathways. This information can serve as roadmap to target genes that are resulting in the difference in fungicidal capabilities within pulmonary murine phagocytes.

Although these studies were conducted in the BALB/c mouse strain, this strain has been well-studied in the model of protection against *C. neoformans* (10, 11, 78, 102). In addition, since we are interested in immune responses, we chose not to use the C57BL/6 model, even though many knock-out mice are available on this background. The C57BL/6 mice are known to have a truncation in CXCL11 (103–105). This truncation is important in *C. neoformans* infections, since previous studies have shown the importance of the STAT-1 signaling pathway (which results in the production of CXCL9, 10, 11) as essential for protective responses to *C. neoformans* (9–11, 13).

To our knowledge, our studies have provided the first examination of individual pulmonary phagocyte subset interactions with *C. neoformans*. The results from these studies lead us to conclude that different populations of murine pulmonary phagocytes interact differently with *C. neoformans*, and these differences depend upon sex of the murine host and differential gene expression, but are not dependent on phagocyte polarization. Future studies using gene knockout mice for pulmonary *C. neoformans* infection will be needed to provide further insight into the functional aspects of genes and signaling pathways identified in these studies. This will guide our studies to eventually target specific genes/pathways for use to prevent intracellular replication and trafficking of *C. neoformans* to the brain in order to prevent meningitis. The results from this study indicate the need for additional research into pulmonary phagocyte subsets and their interactions with intracellular pathogens.

## Data Availability Statement

The original contributions presented in the study are included in the NCBI repository, under Bioproject SRP324663, SRA accessions SRX11181691-SRX11181712 and SRX11522746-SRX11522773 for samples from each subset of cells from both male and female mice, with and without exposure to *C. neoformans.* Further inquiries can be directed to the corresponding author.

## Ethics Statement

This study was carried out in accordance with the recommendations of the Institutional Animal Care and Use Committee at Oklahoma State University.

## Author Contributions

KW designed study, performed statistical analysis, interpreted study results, and participated in drafting and editing of manuscript. AH, BD, and BN assisted in study design, performed experiments and statistical analysis, participated in interpretation of results, and drafted manuscript. AH, BD, and BN performed the experiments. AH and BD analyzed and interpreted the data. AH and KW wrote and edited the manuscript. KW planned and supervised the experiments, data analysis and interpretation, supervised the study and revised the manuscript. All authors contributed to the article and approved the submitted version.

## Funding

This work was supported by research grants P20GM103648 and 1P20GM134973-01 (KLW) from the National Institute of General Medical Sciences of the National Institutes of Health (NIH) and startup funds from Oklahoma State University (KLW). In addition, AH was supported by the National Science Foundation Oklahoma Louis Stokes Alliance for Minority Participation (LSAMP) Bridge to the Doctorate Award # HRD-1612560 and BD was supported by the National Science Foundation LSAMP grant # HRD-1911370 at Oklahoma State University. The content is solely the responsibility of the authors and does not necessarily represent the official views of the funders. The funders had no role in study design, data collection and analysis, decision to publish, or preparation of the manuscript.

## Conflict of Interest

The authors declare that the research was conducted in the absence of any commercial or financial relationships that could be construed as a potential conflict of interest.

## Publisher’s Note

All claims expressed in this article are solely those of the authors and do not necessarily represent those of their affiliated organizations, or those of the publisher, the editors and the reviewers. Any product that may be evaluated in this article, or claim that may be made by its manufacturer, is not guaranteed or endorsed by the publisher.

## References

[1] Kwon-Chung KJ Fraser JA Doering TL Wang Z Janbon G Idnurm A. Cryptococcus Neoformans and Cryptococcus Gattii, the Etiologic Agents of Cryptococcosis. Cold Spring Harb Perspect Med (2014) 4(7):a019760. doi: 10.1101/cshperspect.a019760 24985132 PMC4066639

[2] Ellis DH Pfeiffer TJ. Ecology, Life Cycle, and Infectious Propagule of Cryptococcus Neoformans. Lancet (1990) 336(8720):923–5. doi: 10.1016/0140-6736(90)92283-N 1976940

[3] Perfect JR Casadevall A. Cryptococcosis. Infect Dis Clin N Am (2002) 16(4):837–74. doi: 10.1016/S0891-5520(02)00036-3 12512184

[4] De Leon-Rodriguez CM Rossi DCP Fu MS Dragotakes Q Coelho C Guerrero Ros I. The Outcome of the Cryptococcus Neoformans-Macrophage Interaction Depends on Phagolysosomal Membrane Integrity. J Immunol (2018) 201(2):583–603. doi: 10.4049/jimmunol.1700958 29858266 PMC6245949

[5] Espinosa V Rivera A. First Line of Defense: Innate Cell-Mediated Control of Pulmonary Aspergillosis. Front Microbiol (2016) 7:272. doi: 10.3389/fmicb.2016.00272 26973640 PMC4776213

[6] Margalit A Kavanagh K. The Innate Immune Response to Aspergillus Fumigatus at the Alveolar Surface. FEMS Microbiol Rev (2015) 39(5):670–87. doi: 10.1093/femsre/fuv018 25934117

[7] Cheung DO Halsey K Speert DP. Role of Pulmonary Alveolar Macrophages in Defense of the Lung Against Pseudomonas Aeruginosa. Infect Immun (2000) 68(8):4585–92. doi: 10.1128/IAI.68.8.4585-4592.2000 PMC98382 10899859

[8] Lloyd CM Marsland BJ. Lung Homeostasis: Influence of Age, Microbes, and the Immune System. Immunity (2017) 46(4):549–61. doi: 10.1016/j.immuni.2017.04.005 28423336

[9] Leopold Wager CM Hole CR Wozniak KL Olszewski MA Wormley FL Jr. STAT1 Signaling Is Essential for Protection Against Cryptococcus Neoformans Infection in Mice. J Immunol (2014) 193(8):4060–71. doi: 10.4049/jimmunol.1400318 PMC4185263 25200956

[10] Leopold Wager CM Hole CR Wozniak KL Olszewski MA Mueller M Wormley FL Jr. STAT1 Signaling Within Macrophages is Required for Antifungal Activity Against Cryptococcus Neoformans. Infect Immun (2015) 83(12):4513–27. doi: 10.1128/IAI.00935-15 PMC4645398 26351277

[11] Leopold Wager CM Hole CR Campuzano A Castro-Lopez N Cai H Caballero Van Dyke MC. IFN-Gamma Immune Priming of Macrophages *In Vivo* Induces Prolonged STAT1 Binding and Protection Against Cryptococcus Neoformans. PloS Pathog (2018) 14(10):e1007358. doi: 10.1371/journal.ppat.1007358 30304063 PMC6197699

[12] Hardison SE Ravi S Wozniak KL Young ML Olszewski MA Wormley FL Jr. Pulmonary Infection With an Interferon-Gamma-Producing Cryptococcus Neoformans Strain Results in Classical Macrophage Activation and Protection. Am J Pathol (2010) 176(2):774–85. doi: 10.2353/ajpath.2010.090634 PMC2808084 20056835

[13] Hardison SE Herrera G Young ML Hole CR Wozniak KL Wormley FL Jr. Protective Immunity Against Pulmonary Cryptococcosis is Associated With STAT1-Mediated Classical Macrophage Activation. J Immunol (2012) 189(8):4060–8. doi: 10.4049/jimmunol.1103455 PMC3466339 22984078

[14] Chen GH Teitz-Tennenbaum S Neal LM Murdock BJ Malachowski AN Dils AJ. Local GM-CSF-Dependent Differentiation and Activation of Pulmonary Dendritic Cells and Macrophages Protect Against Progressive Cryptococcal Lung Infection in Mice. J Immunol (2016) 196(4):1810–21. doi: 10.4049/jimmunol.1501512 PMC4744503 26755822

[15] Osterholzer JJ Milam JE Chen GH Toews GB Huffnagle GB Olszewski MA. Role of Dendritic Cells and Alveolar Macrophages in Regulating Early Host Defense Against Pulmonary Infection With Cryptococcus Neoformans. Infect Immun (2009) 77(9):3749–58. doi: 10.1128/IAI.00454-09 PMC2737986 19564388

[16] Wozniak KL Vyas JM Levitz SM. *In Vivo* Role of Dendritic Cells in a Murine Model of Pulmonary Cryptococcosis. Infect Immun (2006) 74(7):3817–24. doi: 10.1128/IAI.00317-06 PMC1489690 16790753

[17] Leopold Wager CM Hole CR Wozniak KL Wormley FL Jr. Cryptococcus and Phagocytes: Complex Interactions That Influence Disease Outcome. Front Microbiol (2016) 7:105. doi: 10.3389/fmicb.2016.00105 26903984 PMC4746234

[18] Wozniak KL Levitz SM. Cryptococcus Neoformans Enters the Endolysosomal Pathway of Dendritic Cells and Is Killed by Lysosomal Components. Infect Immun (2008) 76(10):4764–71. doi: 10.1128/IAI.00660-08 PMC2546838 18678670

[19] Kelly RM Chen J Yauch LE Levitz SM. Opsonic Requirements for Dendritic Cell-Mediated Responses to Cryptococcus Neoformans. Infect Immun (2005) 73(1):592–8. doi: 10.1128/IAI.73.1.592-598.2005 PMC539000 15618199

[20] Levitz SM Farrell TP. Growth Inhibition of Cryptococcus Neoformans by Cultured Human Monocytes: Role of the Capsule, Opsonins, the Culture Surface, and Cytokines. Infect Immun (1990) 58(5):1201–9. doi: 10.1128/iai.58.5.1201-1209.1990 PMC258610 2182538

[21] Bolanos B Mitchell TG. Phagocytosis and Killing of *Cryptococcus Neoformans* by Rat Alveolar Macrophages in the Absence of Serum. J Leukocyte Biol (1989) 46(6):521–8. doi: 10.1002/jlb.46.6.521 2681492

[22] Bolanos B Mitchell TG. Killing of *Cryptococcus Neoformans* by Rat Alveolar Macrophages. J Med Vet Mycol (1989) 27(4):219–28. doi: 10.1080/02681218980000301 2677299

[23] Bolanos B Mitchell TG. Phagocytosis of *Cryptococcus Neoformans* by Rat Alveolar Macrophages. J Med Vet Mycol (1989) 27(4):203–17. doi: 10.1080/02681218980000291 2677298

[24] Weinberg PB Becker S Granger DL Koren HS. Growth Inhibition of Cryptococcus Neoformans by Human Alveolar Macrophages. Am Rev Respir Dis (1987) 136(5):1242–7. doi: 10.1164/ajrccm/136.5.1242 3314617

[25] Coelho C Bocca AL Casadevall A. The Intracellular Life of Cryptococcus Neoformans. Annu Rev Pathol (2014) 9:219–38. doi: 10.1146/annurev-pathol-012513-104653 PMC5127716 24050625

[26] Denham ST Brown JCS. Mechanisms of Pulmonary Escape and Dissemination by Cryptococcus Neoformans. J Fungi (Basel) (2018) 4(1):25–41. doi: 10.3390/jof4010025 PMC5872328 29463005

[27] Feldmesser M Tucker S Casadevall A. Intracellular Parasitism of Macrophages by Cryptococcus Neoformans. Trends Microbiol (2001) 9(6):273–8. doi: 10.1016/S0966-842X(01)02035-2 11390242

[28] Johnston SA May RC. Cryptococcus Interactions With Macrophages: Evasion and Manipulation of the Phagosome by a Fungal Pathogen. Cell Microbiol (2013) 15(3):403–11. doi: 10.1111/cmi.12067 23127124

[29] Levitz SM Tabuni A. Binding of Cryptococcus Neoformans by Human Cultured Macrophages. Requirements for Multiple Complement Receptors and Actin. J Clin Invest (1991) 87(2):528–35. doi: 10.1172/JCI115027 PMC296340 1991837

[30] Levitz SM North EA Jiang Y Nong SH Kornfeld H Harrison TS. Variables Affecting Production of Monocyte Chemotactic Factor 1 From Human Leukocytes Stimulated With Cryptococcus Neoformans. Infect Immun (1997) 65(3):903–8. doi: 10.1128/iai.65.3.903-908.1997 PMC175067 9038295

[31] Feldmesser M Kress Y Novikoff P Casadevall A. Cryptococcus Neoformans Is a Facultative Intracellular Pathogen in Murine Pulmonary Infection. Infect Immun (2000) 68(7):4225–37. doi: 10.1128/IAI.68.7.4225-4237.2000 PMC101732 10858240

[32] Tucker SC Casadevall A. Replication of *Cryptococcus Neoformans* in Macrophages is Accompanied by Phagosomal Permeabilization and Accumulation of Vesicles Containing Polysaccharide in the Cytoplasm. Proc Natl Acad Sci USA (2002) 99(5):3165–70. doi: 10.1073/pnas.052702799 PMC122490 11880650

[33] Alvarez M Casadevall A. Phagosome Extrusion and Host-Cell Survival After Cryptococcus Neoformans Phagocytosis by Macrophages. Curr Biol (2006) 16(21):2161–5. doi: 10.1016/j.cub.2006.09.061 17084702

[34] Johnston SA May RC. The Human Fungal Pathogen Cryptococcus Neoformans Escapes Macrophages by a Phagosome Emptying Mechanism That is Inhibited by Arp2/3 Complex-Mediated Actin Polymerisation. PloS Pathog (2010) 6(8):e1001041. doi: 10.1371/journal.ppat.1001041 20714349 PMC2920849

[35] Charlier C Nielsen K Daou S Brigitte M Chretien F Dromer F. Evidence of a Role for Monocytes in Dissemination and Brain Invasion by Cryptococcus Neoformans. Infect Immun (2009) 77(1):120–7. doi: 10.1128/IAI.01065-08 PMC2612285 18936186

[36] Kechichian TB Shea J Del Poeta M. Depletion of Alveolar Macrophages Decreases the Dissemination of a Glucosylceramide-Deficient Mutant of Cryptococcus Neoformans in Immunodeficient Mice. Infect Immun (2007) 75(10):4792–8. doi: 10.1128/IAI.00587-07 PMC2044542 17664261

[37] Santangelo R Zoellner H Sorrell T Wilson C Donald C Djordjevic J. Role of Extracellular Phospholipases and Mononuclear Phagocytes in Dissemination of Cryptococcosis in a Murine Model. Infect Immun (2004) 72(4):2229–39. doi: 10.1128/IAI.72.4.2229-2239.2004 PMC375158 15039347

[38] Sorrell TC Juillard PG Djordjevic JT Kaufman-Francis K Dietmann A Milonig A. Cryptococcal Transmigration Across a Model Brain Blood-Barrier: Evidence of the Trojan Horse Mechanism and Differences Between Cryptococcus Neoformans Var. Grubii Strain H99 and Cryptococcus Gattii Strain R265. Microbes Infect (2016) 18(1):57–67. doi: 10.1016/j.micinf.2015.08.017 26369713

[39] Gomez Perdiguero E Klapproth K Schulz C Busch K Azzoni E Crozet L. Tissue-Resident Macrophages Originate From Yolk-Sac-Derived Erythro-Myeloid Progenitors. Nature (2015) 518(7540):547–51. doi: 10.1038/nature13989 PMC5997177 25470051

[40] van de Laar L Saelens W De Prijck S Martens L Scott Charlotte L Van Isterdael G. Yolk Sac Macrophages, Fetal Liver, and Adult Monocytes Can Colonize an Empty Niche and Develop Into Functional Tissue-Resident Macrophages. Immunity (2016) 44(4):755–68. doi: 10.1016/j.immuni.2016.02.017 26992565

[41] Davies LC Jenkins SJ Allen JE Taylor PR. Tissue-Resident Macrophages. Nat Immunol (2013) 14(10):986–95. doi: 10.1038/ni.2705 PMC4045180 24048120

[42] Collin M Bigley V. Human Dendritic Cell Subsets: An Update. Immunology (2018) 154(1):3–20. doi: 10.1111/imm.12888 29313948 PMC5904714

[43] Guilliams M De Kleer I Henri S Post S Vanhoutte L De Prijck S. Alveolar Macrophages Develop From Fetal Monocytes That Differentiate Into Long-Lived Cells in the First Week of Life *via* GM-CSF. J Exp Med (2013) 210(10):1977–92. doi: 10.1084/jem.20131199 PMC3782041 24043763

[44] Hoeffel G Chen J Lavin Y Low D Almeida FF See P. C-Myb(+) Erythro-Myeloid Progenitor-Derived Fetal Monocytes Give Rise to Adult Tissue-Resident Macrophages. Immunity (2015) 42(4):665–78. doi: 10.1016/j.immuni.2015.03.011 PMC4545768 25902481

[45] Kopf M Schneider C Nobs SP. The Development and Function of Lung-Resident Macrophages and Dendritic Cells. Nat Immunol (2015) 16(1):36–44. doi: 10.1038/ni.3052 25521683

[46] Epelman S Lavine KJ Randolph GJ. Origin and Functions of Tissue Macrophages. Immunity (2014) 41(1):21–35. doi: 10.1016/j.immuni.2014.06.013 25035951 PMC4470379

[47] Zhang C Yang M Ericsson AC. Function of Macrophages in Disease: Current Understanding on Molecular Mechanisms. Front Immunol (2021) 12:620510. doi: 10.3389/fimmu.2021.620510 33763066 PMC7982479

[48] Satpathy AT Wu X Albring JC Murphy KM. Re(de)fining the Dendritic Cell Lineage. Nat Immunol (2012) 13(12):1145–54. doi: 10.1038/ni.2467 PMC3644874 23160217

[49] Chen GH Olszewski MA McDonald RA Wells JC Paine R 3rd Huffnagle GB. Role of Granulocyte Macrophage Colony-Stimulating Factor in Host Defense Against Pulmonary Cryptococcus Neoformans Infection During Murine Allergic Bronchopulmonary Mycosis. Am J Pathol (2007) 170(3):1028–40. doi: 10.2353/ajpath.2007.060595 PMC1864884 17322386

[50] Liu M Nie H Yang Q He Q Zhang G Li P. Characterization of Pulmonary Interstitial Macrophage Phenotypes in a Mouse Model of Asthma. Med J Wuhan Univ (2014) 35(3):357–61.

[51] Vermaelen K Pauwels R. Accurate and Simple Discrimination of Mouse Pulmonary Dendritic Cell and Macrophage Populations by Flow Cytometry: Methodology and New Insights. Cytometry A (2004) 61(2):170–77. doi: 10.1002/cyto.a.20064 15382026

[52] Gibbings SL Thomas SM Atif SM McCubbrey AL Desch AN Danhorn T. Three Unique Interstitial Macrophages in the Murine Lung at Steady State. Am J Respir Cell Mol Biol (2017) 57(1):66–76. doi: 10.1165/rcmb.2016-0361OC 28257233 PMC5516280

[53] Gautier EL Chow A Spanbroek R Marcelin G Greter M Jakubzick C. Systemic Analysis of PPARgamma in Mouse Macrophage Populations Reveals Marked Diversity in Expression With Critical Roles in Resolution of Inflammation and Airway Immunity. J Immunol (2012) 189(5):2614–24. doi: 10.4049/jimmunol.1200495 PMC3537497 22855714

[54] Tan SY Krasnow MA. Developmental Origin of Lung Macrophage Diversity. Development (2016) 143(8):1318–27. doi: 10.1242/dev.129122 PMC4852511 26952982

[55] Zaynagetdinov R Sherrill TP Kendall PL Segal BH Weller KP Tighe RM. Identification of Myeloid Cell Subsets in Murine Lungs Using Flow Cytometry. Am J Respir Cell Mol Biol (2013) 49(2):180–9. doi: 10.1165/rcmb.2012-0366MA PMC3824033 23492192

[56] Misharin AV Morales-Nebreda L Mutlu GM Budinger GR Perlman H. Flow Cytometric Analysis of Macrophages and Dendritic Cell Subsets in the Mouse Lung. Am J Respir Cell Mol Biol (2013) 49(4):503–10. doi: 10.1165/rcmb.2013-0086MA PMC3824047 23672262

[57] Condon TV Sawyer RT Fenton MJ Riches DW. Lung Dendritic Cells at the Innate-Adaptive Immune Interface. J Leukoc Biol (2011) 90(5):883–95. doi: 10.1189/jlb.0311134 PMC3206474 21807741

[58] Shortman K Liu YJ. Mouse and Human Dendritic Cell Subtypes. Nat Rev Immunol (2002) 2(3):151–61. doi: 10.1038/nri746 11913066

[59] Hey YY Tan JK O’Neill HC. Redefining Myeloid Cell Subsets in Murine Spleen. Front Immunol (2015) 6(652):652. doi: 10.3389/fimmu.2015.00652 26793192 PMC4707843

[60] Nelson BN Hawkins AN Wozniak KL. Pulmonary Macrophage and Dendritic Cell Responses to Cryptococcus Neoformans. Front Cell Infect Microbiol (2020) 10:37. doi: 10.3389/fcimb.2020.00037 32117810 PMC7026008

[61] Kozicky LK Sly LM. Depletion and Reconstitution of Macrophages in Mice. Methods Mol Biol (2019) 1960:101–12. doi: 10.1007/978-1-4939-9167-9_9 30798525

[62] Kirby AC Coles MC Kaye PM. Alveolar Macrophages Transport Pathogens to Lung Draining Lymph Nodes. J Immunol (2009) 183(3):1983–9. doi: 10.4049/jimmunol.0901089 PMC3609601 19620319

[63] Mansour MK Reedy JL Tam JM Vyas JM. Macrophage Cryptococcus Interactions: An Update. Curr Fungal Infect Rep (2014) 8(1):109–15. doi: 10.1007/s12281-013-0165-7 PMC3958962 24660045

[64] Voelz K May RC. Cryptococcal Interactions With the Host Immune System. Eukaryot Cell (2010) 6:835–46. doi: 10.1128/EC.00039-10 PMC2901644 20382758

[65] Chamberlain LM Godek ML Gonzalez-Juarrero M Grainger DW. Phenotypic non-Equivalence of Murine (Monocyte-) Macrophage Cells in Biomaterial and Inflammatory Models. J Biomed Materials Res Part A (2009) 88A(4):858–71. doi: 10.1002/jbm.a.31930 PMC10031642 18357567

[66] Voelz K Lammas DA May RC. Cytokine Signaling Regulates the Outcome of Intracellular Macrophage Parasitism by Cryptococcus Neoformans. Infect Immun (2009) 77(8):3450–7. doi: 10.1128/IAI.00297-09 PMC2715691 19487474

[67] Garelnabi M Taylor-Smith LM Bielska E Hall RA Stones D May RC. Quantifying Donor-to-Donor Variation in Macrophage Responses to the Human Fungal Pathogen Cryptococcus Neoformans. PloS One (2018) 13(3):e0194615. doi: 10.1371/journal.pone.0194615 29596441 PMC5875765

[68] Patel VI Booth JL Duggan ES Cate S White VL Hutchings D. Transcriptional Classification and Functional Characterization of Human Airway Macrophage and Dendritic Cell Subsets. J Immunol (2017) 198(3):1183–201. doi: 10.4049/jimmunol.1600777 PMC5262539 28031342

[69] Huang L Nazarova EV Tan S Liu Y Russell DG. Growth of Mycobacterium Tuberculosis *In Vivo* Segregates With Host Macrophage Metabolism and Ontogeny. J Exp Med (2018) 215(4):1135–52. doi: 10.1084/jem.20172020 PMC5881470 29500179

[70] Huang L Nazarova EV Russell DG. Mycobacterium Tuberculosis: Bacterial Fitness Within the Host Macrophage. Microbiol Spectr (2019) 7(2):1–12. doi: 10.1128/microbiolspec.BAI-0001-2019 PMC6459685 30848232

[71] Murray PJ. Immune Regulation by Monocytes. Semin Immunol (2018) 35:12–8. doi: 10.1016/j.smim.2017.12.005 29290545

[72] Traynor TR Kuziel WA Toews GB Huffnagle GB. CCR2 Expression Determines T1 *Versus* T2 Polarization During Pulmonary *Cryptococcus Neoformans* Infection. J Immunol (2000) 164(4):2021–7. doi: 10.4049/jimmunol.164.4.2021 10657654

[73] Osterholzer JJ Curtis JL Polak T Ames T Chen GH McDonald R. CCR2 Mediates Conventional Dendritic Cell Recruitment and the Formation of Bronchovascular Mononuclear Cell Infiltrates in the Lungs of Mice Infected With Cryptococcus Neoformans. J Immunol (2008) 181(1):610–20. doi: 10.4049/jimmunol.181.1.610 PMC2735104 18566428

[74] Osterholzer JJ Surana R Milam JE Montano GT Chen GH Sonstein J. Cryptococcal Urease Promotes the Accumulation of Immature Dendritic Cells and a non-Protective T2 Immune Response Within the Lung. Am J Pathol (2009) 174(3):932–43. doi: 10.2353/ajpath.2009.080673 PMC2665753 19218345

[75] Osterholzer JJ Chen GH Olszewski MA Zhang YM Curtis JL Huffnagle GB. Chemokine Receptor 2-Mediated Accumulation of Fungicidal Exudate Macrophages in Mice That Clear Cryptococcal Lung Infection. Am J Pathol (2011) 178(1):198–211. doi: 10.1016/j.ajpath.2010.11.006 21224057 PMC3069860

[76] Heung LJ Hohl TM. Inflammatory Monocytes are Detrimental to the Host Immune Response During Acute Infection With Cryptococcus Neoformans. PloS Pathog (2019) 15(3):e1007627. doi: 10.1371/journal.ppat.1007627 30897162 PMC6428256

[77] Wozniak KL. Interactions of Cryptococcus With Dendritic Cells. J Fungi (Basel) (2018) 4(1):36–43. doi: 10.3390/jof4010036 PMC5872339 29543719

[78] Wozniak KL Ravi S Macias S Young ML Olszewski MA Steele C. Insights Into the Mechanisms of Protective Immunity Against *Cryptococcus Neoformans* Infection Using a Mouse Model of Pulmonary Cryptococcosis. PloS One (2009) 4(9):e6854. doi: 10.1371/journal.pone.0006854 19727388 PMC2731172

[79] Gibbings SL Jakubzick CV. Isolation and Characterization of Mononuclear Phagocytes in the Mouse Lung and Lymph Nodes. Methods Mol Biol (2018) 1809:33–44. doi: 10.1007/978-1-4939-8570-8_3 29987780 PMC6262824

[80] Sung SS Fu SM Rose CE Jr. Gaskin F Ju ST Beaty SR. A Major Lung CD103 (Alphae)-Beta7 Integrin-Positive Epithelial Dendritic Cell Population Expressing Langerin and Tight Junction Proteins. J Immunol (2006) 176(4):2161–72. doi: 10.4049/jimmunol.176.4.2161 16455972

[81] Hole CR Bui H Wormley FL Jr. Wozniak KL. Mechanisms of Dendritic Cell Lysosomal Killing of Cryptococcus. Sci Rep (2012) 2:739. doi: 10.1038/srep00739 23074646 PMC3472389

[82] Nelson BN Beakley SG Posey S Conn B Maritz E Seshu J. Antifungal Activity of Dendritic Cell Lysosomal Proteins Against Cryptococcus Neoformans. Sci Rep (2021) 11(1):13619. doi: 10.1038/s41598-021-92991-6 34193926 PMC8245489

[83] Rajasingham R Smith RM Park BJ Jarvis JN Govender NP Chiller TM. Global Burden of Disease of HIV-Associated Cryptococcal Meningitis: An Updated Analysis. Lancet Infect Dis (2017) 17(8):873–81. doi: 10.1016/S1473-3099(17)30243-8 PMC5818156 28483415

[84] Subramani A Griggs P Frantzen N Mendez J Tucker J Murriel J. Intracellular Cryptococcus Neoformans Disrupts the Transcriptome Profile of M1- and M2-Polarized Host Macrophages. PloS One (2020) 15(8):e0233818. doi: 10.1371/journal.pone.0233818 32857777 PMC7454990

[85] Hoffmann FM Berger JL Lingel I Laumonnier Y Lewkowich IP Schmudde I. Distribution and Interaction of Murine Pulmonary Phagocytes in the Naive and Allergic Lung. Front Immunol (2018) 9:1046. doi: 10.3389/fimmu.2018.01046 PMC5964136 29868009

[86] Gordon S Pluddemann A Martinez Estrada F. Macrophage Heterogeneity in Tissues: Phenotypic Diversity and Functions. Immunol Rev (2014) 262(1):36–55. doi: 10.1111/imr.12223 25319326 PMC4231239

[87] Serbina NV Jia T Hohl TM Pamer EG. Monocyte-Mediated Defense Against Microbial Pathogens. Annu Rev Immunol (2008) 26:421–52. doi: 10.1146/annurev.immunol.26.021607.090326 PMC2921669 18303997

[88] Pirofski LA Casadevall A. The State of Latency in Microbial Pathogenesis. J Clin Invest (2020) 130(9):4525–31. doi: 10.1172/JCI136221 PMC7456213 32804154

[89] Pisu D Huang L Grenier JK Russell DG. Dual RNA-Seq of Mtb-Infected Macrophages *In Vivo* Reveals Ontologically Distinct Host-Pathogen Interactions. Cell Rep (2020) 30(2):335–50 e4. doi: 10.1016/j.celrep.2019.12.033 31940480 PMC7032562

[90] Guess TE Rosen JA McClelland EE. An Overview of Sex Bias in C. Neoformans Infections. J Fungi (Basel) (2018) 4(2):49–59. doi: 10.3390/jof4020049 PMC6023476 29670032

[91] Ramachandran P Pellicoro A Vernon MA Boulter L Aucott RL Ali A. Differential Ly-6C Expression Identifies the Recruited Macrophage Phenotype, Which Orchestrates the Regression of Murine Liver Fibrosis. Proc Natl Acad Sci USA (2012) 109(46):E3186–95. doi: 10.1073/pnas.1119964109 PMC3503234 23100531

[92] Butenko S Satyanarayanan SK Assi S Schif-Zuck S Sher N Ariel A. Transcriptomic Analysis of Monocyte-Derived Non-Phagocytic Macrophages Favors a Role in Limiting Tissue Repair and Fibrosis. Front Immunol (2020) 11:405. doi: 10.3389/fimmu.2020.00405 32296415 PMC7136412

[93] Zhang Y Wang F Tompkins KC McNamara A Jain AV Moore BB. Robust Th1 and Th17 Immunity Supports Pulmonary Clearance But Cannot Prevent Systemic Dissemination of Highly Virulent Cryptococcus Neoformans H99. Am J Pathol (2009) 175(6):2489–500. doi: 10.2353/ajpath.2009.090530 PMC2789623 19893050

[94] He X Lyons DM Toffaletti DL Wang F Qiu Y Davis MJ. Virulence Factors Identified by Cryptococcus Neoformans Mutant Screen Differentially Modulate Lung Immune Responses and Brain Dissemination. Am J Pathol (2012) 181(4):1356–66. doi: 10.1016/j.ajpath.2012.06.012 PMC3463625 22846723

[95] Muller U Stenzel W Kohler G Polte T Blessing M Mann A. A Gene-Dosage Effect for Interleukin-4 Receptor Alpha-Chain Expression has an Impact on Th2-Mediated Allergic Inflammation During Bronchopulmonary Mycosis. J Infect Dis (2008) 198(11):1714–21. doi: 10.1086/593068 18954266

[96] Hewitt EW. The MHC Class I Antigen Presentation Pathway: Strategies for Viral Immune Evasion. Immunology (2003) 110(2):163–9. doi: 10.1046/j.1365-2567.2003.01738.x PMC1783040 14511229

[97] Muntjewerff EM Meesters LD van den Bogaart G Revelo NH. Reverse Signaling by MHC-I Molecules in Immune and Non-Immune Cell Types. Front Immunol (2020) 11(3264):605958. doi: 10.3389/fimmu.2020.605958 33384693 PMC7770133

[98] Embgenbroich M Burgdorf S. Current Concepts of Antigen Cross-Presentation. Front Immunol (2018) 9:1643. doi: 10.3389/fimmu.2018.01643 30061897 PMC6054923

[99] Cruz FM Colbert JD Merino E Kriegsman BA Rock KL. The Biology and Underlying Mechanisms of Cross-Presentation of Exogenous Antigens on MHC-I Molecules. Annu Rev Immunol (2017) 35:149–76. doi: 10.1146/annurev-immunol-041015-055254 PMC5508990 28125356

[100] Wieczorek M Abualrous ET Sticht J Alvaro-Benito M Stolzenberg S Noe F. Major Histocompatibility Complex (MHC) Class I and MHC Class II Proteins: Conformational Plasticity in Antigen Presentation. Front Immunol (2017) 8:292. doi: 10.3389/fimmu.2017.00292 28367149 PMC5355494

[101] Gutierrez-Martinez E Planes R Anselmi G Reynolds M Menezes S Adiko AC. Cross-Presentation of Cell-Associated Antigens by MHC Class I in Dendritic Cell Subsets. Front Immunol (2015) 6:363. doi: 10.3389/fimmu.2015.00363 26236315 PMC4505393

[102] Hole CR Wager CML Castro-Lopez N Campuzano A Cai H Wozniak KL. Induction of Memory-Like Dendritic Cell Responses *In Vivo* . Nat Commun (2019) 10(1):2955. doi: 10.1038/s41467-019-10486-5 31273203 PMC6609631

[103] Sellers RS Clifford CB Treuting PM Brayton C. Immunological Variation Between Inbred Laboratory Mouse Strains: Points to Consider in Phenotyping Genetically Immunomodified Mice. Vet Pathol (2012) 49(1):32–43. doi: 10.1177/0300985811429314 22135019

[104] Winkler AE Brotman JJ Pittman ME Judd NP Lewis JS Jr. Schreiber RD. CXCR3 Enhances a T-Cell-Dependent Epidermal Proliferative Response and Promotes Skin Tumorigenesis. Cancer Res (2011) 71(17):5707–16. doi: 10.1158/0008-5472.CAN-11-0907 PMC3165086 21734014

[105] Sierro F Biben C Martinez-Munoz L Mellado M Ransohoff RM Li M. Disrupted Cardiac Development But Normal Hematopoiesis in Mice Deficient in the Second CXCL12/SDF-1 Receptor, CXCR7. Proc Natl Acad Sci USA (2007) 104(37):14759–64. doi: 10.1073/pnas.0702229104 PMC1976222 17804806

