# Supplementary material for: Transcriptional Changes in Pulmonary Phagocyte Subsets Dictate the Outcome Following Interaction With The Fungal Pathogen Cryptococcus neoformans
Source: Front Immunol. 2021 Sep 28;12:722500. doi: 10.3389/fimmu.2021.722500 (PMC8505728; doi:10.3389/fimmu.2021.722500)
Supplement: Supplementary file 1 [file Table_1.docx]

**Supplementary Table 1.**

| **Cell Type** | **Sex** | **Gene Name** | **log2(fold change)** | **p-value** |
| --- | --- | --- | --- | --- |
| **AM** | **Male** | Gm25679 | 7.030692 | 1.43E-14 |
|  |  | Cmtr1 | 6.770932 | 0.002864 |
|  |  | Gm16174 | 6.316446 | 0.014079 |
|  |  | Gm15417 | 6.126385 | 0.0219 |
|  |  | Unc5c | 6.126385 | 0.0219 |
|  |  | Nedd4 | 6.126385 | 0.0219 |
|  |  | A530017D24Rik | 6.126385 | 0.0219 |
|  |  | Mtcl1 | 6.126385 | 0.0219 |
|  |  | Gm43774 | 5.90743 | 0.034783 |
|  |  | Tnfrsf9 | 5.90743 | 0.034783 |
|  |  | Prr15 | 5.90743 | 0.034783 |
|  |  | 9330179D12Rik | 5.90743 | 0.034783 |
|  |  | Gm9905 | 5.90743 | 0.034783 |
|  |  | Gng7 | 5.90743 | 0.034783 |
|  |  | 5830454E08Rik | 5.90743 | 0.034783 |
|  |  | Sptbn2 | -5.77949 | 0.034783 |
|  |  | Padi2 | -5.94649 | 0.0219 |
|  |  | 4933440N22Rik | -5.94649 | 0.0219 |
|  |  | Kif5a | -5.94649 | 0.0219 |
|  |  | Dnase1l3 | -5.94649 | 0.0219 |
|  |  | Areg | -6.09616 | 0.014079 |
|  |  | Thap2 | -6.09616 | 0.014079 |
|  |  | Gm11496 | -6.09616 | 0.014079 |
|  |  | 2610037D02Rik | -6.09616 | 0.014079 |
|  |  | Tnfsf15 | -6.23174 | 0.014079 |
|  |  | Gm43323 | -6.35567 | 0.009224 |
|  |  | Gm47260 | -6.35567 | 0.009224 |
|  |  | Zfp69 | -6.46979 | 0.006149 |
|  |  | Gm12366 | -6.85298 | 0.001996 |
|  |  | Gm11336 | -6.85298 | 0.001996 |
| **AM** | **Female** | Gm47518 | 7.649719 | 7.45E-05 |
|  |  | Gm15726 | 7.091257 | 0.000727 |
|  |  | Gm24830 | 6.870627 | 0.001996 |
|  |  | Gm12791 | 6.511575 | 0.006149 |
|  |  | Gm45552 | 6.291815 | 0.009224 |
|  |  | Tpbg | 6.167958 | 0.014079 |
|  |  | Gm17068 | 6.167958 | 0.014079 |
|  |  | Gm20632 | 6.032459 | 0.0219 |
|  |  | Rpl6l | 6.032459 | 0.0219 |
|  |  | Gm47234 | 6.032459 | 0.0219 |
|  |  | Gm18588 | 5.882903 | 0.034783 |
|  |  | Tnfsf15 | 5.882903 | 0.034783 |
|  |  | Mettl27 | 5.882903 | 0.034783 |
|  |  | Gm44153 | 5.882903 | 0.034783 |
|  |  | Gm40645 | 5.882903 | 0.034783 |
|  |  | Gm45805 | -6.00571 | 0.034783 |
|  |  | Gm23444 | -6.00571 | 0.034783 |
|  |  | Ptprn2 | -6.00571 | 0.034783 |
|  |  | Mov10l1 | -6.00571 | 0.034783 |
|  |  | Mpv17l | -6.00571 | 0.034783 |
|  |  | Fbn2 | -6.00571 | 0.034783 |
|  |  | Unc80 | -6.22489 | 0.0219 |
|  |  | Igfn1 | -6.22489 | 0.0219 |
|  |  | Gm13608 | -6.22489 | 0.0219 |
|  |  | Adamtsl1 | -6.22489 | 0.0219 |
|  |  | Gm8797 | -6.41512 | 0.009224 |
|  |  | Efcab11 | -6.41512 | 0.009224 |
|  |  | H2-Q10 | -6.41512 | 0.009224 |
|  |  | Nanos1 | -6.58317 | 0.006149 |
|  |  | Gm7638 | -8.30956 | 5.19E-06 |
| **IM** | **Male** | Gm15908 | 6.996 | 0.000194 |
|  |  | Scnn1g | 6.805 | 0.000517 |
|  |  | Aqp3 | 6.805 | 0.000517 |
|  |  | Gprc5d | 6.805 | 0.000517 |
|  |  | Ntn5 | 6.805 | 0.000517 |
|  |  | Gm13226 | 6.805 | 0.000517 |
|  |  | Slc16a8 | 6.699 | 0.000858 |
|  |  | Bcas3os1 | 6.699 | 0.000858 |
|  |  | Oit3 | 6.5847 | 0.001437 |
|  |  | Wdr20rt | 6.5847 | 0.001437 |
|  |  | Gm11841 | 6.5847 | 0.001437 |
|  |  | Tomm6os | 6.5847 | 0.001437 |
|  |  | Gm12348 | 6.5847 | 0.001437 |
|  |  | 2810425M01Rik | 6.5847 | 0.001437 |
|  |  | Gabrr2 | 6.4605 | 0.002435 |
|  |  | Lrrc16b | -6.7296 | 0.000858 |
|  |  | 4931409K22Rik | -6.7296 | 0.000858 |
|  |  | Rpl13-ps3 | -6.7296 | 0.000858 |
|  |  | Gm13443 | -6.7296 | 0.000858 |
|  |  | Gm13511 | -6.7296 | 0.000858 |
|  |  | Gm26771 | -6.7296 | 0.000858 |
|  |  | RP23-451E8.1 | -6.7296 | 0.000858 |
|  |  | Acvr1c | -6.8356 | 0.000517 |
|  |  | Gm9754 | -6.9343 | 0.000315 |
|  |  | Dmbt1 | -7.0266 | 0.000194 |
|  |  | Gm10653 | -7.0266 | 0.000194 |
|  |  | Nynrin | -7.1953 | 7.55E-05 |
|  |  | Gpr113 | -7.2728 | 4.78E-05 |
|  |  | 9930004E17Rik | -7.5468 | 8.28E-06 |
|  |  | Hspb9 | -7.7228 | 2.40E-06 |
| **IM** | **Female** | Rnu1a1 | 8.530398 | 1.35E-20 |
|  |  | Gm7638 | 7.955085 | 2.82E-05 |
|  |  | Gm12428 | 6.191527 | 0.0219 |
|  |  | Gm9889 | 6.191527 | 0.0219 |
|  |  | Gm22154 | 6.191527 | 0.0219 |
|  |  | Gm22973 | 6.191527 | 0.0219 |
|  |  | Gm6430 | 5.972421 | 0.034783 |
|  |  | Kctd19 | 5.972421 | 0.034783 |
|  |  | Gm48766 | 5.972421 | 0.034783 |
|  |  | Pnma1 | 5.972421 | 0.034783 |
|  |  | Gm25679 | 5.877594 | 5.86E-11 |
|  |  | mt-Rnr1 | 3.873328 | 1.22E-07 |
|  |  | Ftl2-ps | 3.797119 | 1.96E-06 |
|  |  | Mycl | 3.635049 | 0.027969 |
|  |  | Tmem100 | 3.635049 | 0.027969 |
|  |  | 2010310C07Rik | -6.05321 | 0.0219 |
|  |  | Zfp93 | -6.05321 | 0.0219 |
|  |  | Gm45094 | -6.05321 | 0.0219 |
|  |  | Tjp3 | -6.05321 | 0.0219 |
|  |  | C130012C08Rik | -6.05321 | 0.0219 |
|  |  | Notch3 | -6.05321 | 0.0219 |
|  |  | Aldh3b3 | -6.05321 | 0.0219 |
|  |  | Zfp300 | -6.18874 | 0.014079 |
|  |  | Pidd1 | -6.18874 | 0.014079 |
|  |  | Sycp3 | -6.18874 | 0.014079 |
|  |  | A130051J06Rik | -6.18874 | 0.014079 |
|  |  | Prss36 | -6.4267 | 0.006149 |
|  |  | Scin | -6.53242 | 0.006149 |
|  |  | Frat1 | -6.72312 | 0.002864 |
|  |  | Hexdc | -7.52461 | 0.000126 |
| **Ly6c-** | **Male** | Vti1a | 12.556 | 5.08E-31 |
|  |  | Mettl2 | 12.139 | 4.39E-28 |
|  |  | Acot7 | 12.009 | 3.55E-27 |
|  |  | Tma16 | 11.995 | 4.28E-27 |
|  |  | Dut | 11.991 | 4.70E-27 |
|  |  | Zfp608 | 11.989 | 4.92E-27 |
|  |  | Angpt2 | 11.962 | 7.54E-27 |
|  |  | Gpr108 | 11.948 | 9.14E-27 |
|  |  | Robo2 | 11.709 | 4.04E-25 |
|  |  | Tcaim | 11.701 | 4.52E-25 |
|  |  | Stx2 | 11.646 | 1.07E-24 |
|  |  | Esrp2 | 11.623 | 1.52E-24 |
|  |  | Sfmbt2 | 11.617 | 1.71E-24 |
|  |  | Chml | 11.54 | 5.43E-24 |
|  |  | Ccnf | 11.528 | 6.54E-24 |
|  |  | Neu1 | -11.528 | 4.00E-26 |
|  |  | Chst14 | -11.528 | 4.00E-26 |
|  |  | Crtc3 | -11.534 | 3.75E-26 |
|  |  | Tab1 | -11.55 | 2.90E-26 |
|  |  | Galnt12 | -11.565 | 2.25E-26 |
|  |  | 3110057O12Rik | -11.617 | 1.00E-26 |
|  |  | Eid2 | -11.637 | 6.96E-27 |
|  |  | Wdr90 | -11.637 | 6.96E-27 |
|  |  | Vps18 | -11.657 | 5.16E-27 |
|  |  | Pccb | -11.854 | 2.14E-28 |
|  |  | Wdr55 | -11.859 | 2.03E-28 |
|  |  | Cd36 | -12.039 | 1.06E-29 |
|  |  | Batf | -12.069 | 6.67E-30 |
|  |  | Plcb2 | -12.106 | 3.55E-30 |
|  |  | Hist4h4 | -12.109 | 3.39E-30 |
| **Ly6c-** | **Female** | Gm26232 | 7.834585 | 3.57E-05 |
|  |  | 1700065I16Rik | 7.78038 | 4.53E-05 |
|  |  | Gm31786 | 7.724059 | 5.79E-05 |
|  |  | Frmd7 | 7.403836 | 0.000218 |
|  |  | Gm16150 | 7.252779 | 0.000391 |
|  |  | A530032D15Rik | 7.170873 | 0.00053 |
|  |  | Gm43457 | 7.170873 | 0.00053 |
|  |  | Prss35 | 7.170873 | 0.00053 |
|  |  | Gm37962 | 7.170873 | 0.00053 |
|  |  | Gm14636 | 7.084036 | 0.000727 |
|  |  | Vmn1r43 | 7.084036 | 0.000727 |
|  |  | Qprt | 7.084036 | 0.000727 |
|  |  | Frmd8os | 7.084036 | 0.000727 |
|  |  | Asb9 | 6.991636 | 0.001006 |
|  |  | Cd300e | 6.991636 | 0.001006 |
|  |  | Gm15851 | -6.84964 | 0.001996 |
|  |  | Slc10a5 | -6.84964 | 0.001996 |
|  |  | Gm43312 | -6.84964 | 0.001996 |
|  |  | Gm32996 | -6.84964 | 0.001996 |
|  |  | Vsnl1 | -6.94197 | 0.001409 |
|  |  | Gm20150 | -7.02874 | 0.001006 |
|  |  | A230001M10Rik | -7.11059 | 0.000727 |
|  |  | Gm3164 | -7.18804 | 0.00053 |
|  |  | Gm48111 | -7.18804 | 0.00053 |
|  |  | Flywch2 | -7.18804 | 0.00053 |
|  |  | Gm31166 | -7.18804 | 0.00053 |
|  |  | Erv3 | -7.26155 | 0.000391 |
|  |  | Aplnr | -7.33149 | 0.000291 |
|  |  | Slc16a13 | -7.3982 | 0.000218 |
|  |  | Pelo | -8.39391 | 2.05E-06 |
| **Ly6c+** | **Male** | Pck1 | 7.215113 | 0.00053 |
|  |  | 1700020N18Rik | 7.122651 | 0.000727 |
|  |  | Spc24 | 7.122651 | 0.000727 |
|  |  | Gm43721 | 6.917791 | 0.001409 |
|  |  | D730001G18Rik | 6.917791 | 0.001409 |
|  |  | Asb18 | 6.803308 | 0.002864 |
|  |  | Gm26771 | 6.803308 | 0.002864 |
|  |  | Gm9758 | 6.803308 | 0.002864 |
|  |  | Gm5987 | 6.803308 | 0.002864 |
|  |  | Gsdma | 6.803308 | 0.002864 |
|  |  | Apob | 6.803308 | 0.002864 |
|  |  | Cpn1 | 6.803308 | 0.002864 |
|  |  | Ankub1 | 6.678951 | 0.004166 |
|  |  | Psg17 | 6.678951 | 0.004166 |
|  |  | Gm37425 | 6.678951 | 0.004166 |
|  |  | Mzt2 | -6.42214 | 0.006149 |
|  |  | H2-Ob | -6.42214 | 0.006149 |
|  |  | Gm10143 | -6.42214 | 0.006149 |
|  |  | Prg3 | -6.53632 | 0.004166 |
|  |  | Gm26818 | -6.53632 | 0.004166 |
|  |  | Trav7d-4 | -6.53632 | 0.004166 |
|  |  | Gm43504 | -6.64213 | 0.004166 |
|  |  | Gm2245 | -6.64213 | 0.004166 |
|  |  | Recql4 | -6.64213 | 0.004166 |
|  |  | Tekt5 | -6.7407 | 0.002864 |
|  |  | Mapkapk5 | -6.83297 | 0.001996 |
|  |  | Car7 | -6.83297 | 0.001996 |
|  |  | Iglc3 | -6.83297 | 0.001996 |
|  |  | Snhg3 | -6.91968 | 0.001409 |
|  |  | Snx22 | -7.35267 | 0.000291 |
| **Ly6c+** | **Female** | Gm26444 | 7.466844 | 0.004166 |
|  |  | Gm23472 | 7.275363 | 0.006149 |
|  |  | Gm23136 | 7.054522 | 0.009224 |
|  |  | Gm22317 | 7.054522 | 0.009224 |
|  |  | Gm8000 | 7.054522 | 0.009224 |
|  |  | Gm7887 | 6.793656 | 0.0219 |
|  |  | Gm26944 | 6.793656 | 0.0219 |
|  |  | Zfp469 | 6.793656 | 0.0219 |
|  |  | Gm12187 | 6.793656 | 0.0219 |
|  |  | Lpo | 6.793656 | 0.0219 |
|  |  | Gm23971 | 5.766519 | 5.38E-12 |
|  |  | Gm25679 | 5.353728 | 4.28E-06 |
|  |  | Rn7s6 | 5.183102 | 7.11E-08 |
|  |  | Lars2 | 5.069199 | 6.79E-11 |
|  |  | Tpm1 | 4.914513 | 4.97E-10 |
|  |  | Cbx8 | -7.10753 | 0.010215 |
|  |  | Hsd3b7 | -7.1578 | 0.010215 |
|  |  | E2f8 | -7.20637 | 0.007447 |
|  |  | Ftl2-ps | -7.29887 | 0.005479 |
|  |  | Chaf1b | -7.34298 | 0.005479 |
|  |  | Alkbh8 | -7.38579 | 0.004066 |
|  |  | Pik3ip1 | -7.38579 | 0.004066 |
|  |  | Ddx11 | -7.38579 | 0.004066 |
|  |  | Sptbn2 | -7.38579 | 0.004066 |
|  |  | Pclaf | -7.46777 | 0.003043 |
|  |  | Ammecr1 | -7.50708 | 0.003043 |
|  |  | H2-T24 | -7.58263 | 0.002295 |
|  |  | Hist1h2ab | -7.65441 | 0.001744 |
|  |  | E2f6 | -7.81964 | 0.000797 |
|  |  | Hydin | -7.9104 | 0.000622 |
| **CD11b+** | **Male** | Ighv4-1 | 9.1396 | 3.82E-12 |
|  |  | Igkv6-13 | 8.3185 | 1.51E-08 |
|  |  | Gm12064 | 7.8926 | 7.40E-07 |
|  |  | Adc | 7.8422 | 1.09E-06 |
|  |  | Slc5a10 | 7.79 | 1.61E-06 |
|  |  | Gm12372 | 7.79 | 1.61E-06 |
|  |  | Mid2 | 7.7358 | 2.40E-06 |
|  |  | Sel1l3 | 7.6795 | 3.61E-06 |
|  |  | Krt26 | 7.6795 | 3.61E-06 |
|  |  | Gm13006 | 7.6795 | 3.61E-06 |
|  |  | Gucy1b3 | 7.6208 | 5.45E-06 |
|  |  | Prdm16 | 7.6208 | 5.45E-06 |
|  |  | Ccnjl | 7.5598 | 8.28E-06 |
|  |  | Lrrn4cl | 7.5598 | 8.28E-06 |
|  |  | Trbv13-2 | 7.5598 | 8.28E-06 |
|  |  | Lox | -7.4398 | 1.27E-05 |
|  |  | Fndc1 | -7.4398 | 1.27E-05 |
|  |  | Ppm1e | -7.5035 | 8.28E-06 |
|  |  | Gja1 | -7.5035 | 8.28E-06 |
|  |  | Gm12276 | -7.5035 | 8.28E-06 |
|  |  | Gm20508 | -7.5035 | 8.28E-06 |
|  |  | Gm26916 | -7.5035 | 8.28E-06 |
|  |  | Snph | -7.5646 | 5.45E-06 |
|  |  | Guca1a | -7.6232 | 3.61E-06 |
|  |  | 1700101I11Rik | -7.6232 | 3.61E-06 |
|  |  | Mc1r | -7.6795 | 2.40E-06 |
|  |  | 1110019D14Rik | -7.6795 | 2.40E-06 |
|  |  | Gm14834 | -7.7859 | 1.09E-06 |
|  |  | Itpka | -7.8363 | 7.40E-07 |
|  |  | Lingo3 | -8.022 | 2.40E-07 |
| **CD11b+** | **Female** | Igkv6-32 | 7.35114 | 0.00053 |
|  |  | 4933424N20Rik | 6.869244 | 0.002864 |
|  |  | Hspb1 | 6.869244 | 0.002864 |
|  |  | Gm37945 | 6.869244 | 0.002864 |
|  |  | Bicdl2 | 6.869244 | 0.002864 |
|  |  | Gm49495 | 6.718611 | 0.004166 |
|  |  | Nt5c1a | 6.550397 | 0.009224 |
|  |  | Rasl12 | 6.550397 | 0.009224 |
|  |  | AC165079.1 | 6.550397 | 0.009224 |
|  |  | Gm28411 | 6.359949 | 0.014079 |
|  |  | Crct1 | 6.359949 | 0.014079 |
|  |  | Gm35315 | 6.359949 | 0.014079 |
|  |  | Dync1i1 | 6.359949 | 0.014079 |
|  |  | Gm43879 | 6.359949 | 0.014079 |
|  |  | Vmn1r26 | 6.359949 | 0.014079 |
|  |  | Glis3 | -6.32914 | 0.009224 |
|  |  | Adora1 | -6.43478 | 0.006149 |
|  |  | Gm6451 | -6.43478 | 0.006149 |
|  |  | Gm20560 | -6.43478 | 0.006149 |
|  |  | Gpr19 | -6.43478 | 0.006149 |
|  |  | Cbx3-ps6 | -6.43478 | 0.006149 |
|  |  | A630072L19Rik | -6.43478 | 0.006149 |
|  |  | Ccdc154 | -6.43478 | 0.006149 |
|  |  | Etl4 | -6.5332 | 0.006149 |
|  |  | Tgm5 | -6.5332 | 0.006149 |
|  |  | Sim1 | -6.5332 | 0.006149 |
|  |  | Celf6 | -6.5332 | 0.006149 |
|  |  | Gm12905 | -6.79364 | 0.001996 |
|  |  | Igkv8-30 | -7.08078 | 0.000727 |
|  |  | Igkv5-43 | -7.52539 | 0.000165 |
| **CD103+** | **Male** | Kmo | 11.709 | 1.72E-26 |
|  |  | Cadps2 | 11.201 | 4.46E-23 |
|  |  | Zfp40 | 10.894 | 4.45E-21 |
|  |  | Il12a | 10.872 | 6.40E-21 |
|  |  | Gm16147 | 10.774 | 2.63E-20 |
|  |  | Psmd10 | 10.681 | 1.06E-19 |
|  |  | Igkv14-126 | 10.662 | 1.45E-19 |
|  |  | Fam171a1 | 10.555 | 6.46E-19 |
|  |  | Gm9889 | 10.519 | 1.13E-18 |
|  |  | Pstk | 10.498 | 1.41E-18 |
|  |  | Osgepl1 | 10.476 | 1.99E-18 |
|  |  | Slc19a1 | 10.337 | 1.35E-17 |
|  |  | Igkj4 | 10.288 | 2.89E-17 |
|  |  | Vmac | 10.271 | 1.88E-18 |
|  |  | Eci1 | 10.237 | 2.80E-18 |
|  |  | Akirin1-ps | -9.3664 | 3.28E-13 |
|  |  | Gm13651 | -9.3664 | 3.28E-13 |
|  |  | Slc22a18 | -9.4048 | 2.15E-13 |
|  |  | Gm10134 | -9.4048 | 2.15E-13 |
|  |  | Slc39a12 | -9.4236 | 1.75E-13 |
|  |  | Fscn2 | -9.4422 | 1.42E-13 |
|  |  | Abhd3 | -9.4605 | 9.45E-14 |
|  |  | RP23-124D10.4 | -9.5142 | 5.19E-14 |
|  |  | Lin28b | -9.5317 | 4.26E-14 |
|  |  | Gm8482 | -9.8002 | 1.33E-15 |
|  |  | Gm14537 | -9.8002 | 1.33E-15 |
|  |  | Gm13625 | -9.8287 | 9.45E-16 |
|  |  | 44259 | -10.469 | 1.38E-19 |
|  |  | Gdf15 | -10.695 | 5.03E-21 |
|  |  | Il23a | -11.956 | 1.61E-29 |
| **CD103+** | **Female** | Gm29488 | 6.371356 | 0.009224 |
|  |  | Insrr | 6.371356 | 0.009224 |
|  |  | Gm12679 | 6.371356 | 0.009224 |
|  |  | Fbxo41 | 6.371356 | 0.009224 |
|  |  | Fam161b | 6.371356 | 0.009224 |
|  |  | Sco2 | 6.371356 | 0.009224 |
|  |  | AC167363.1 | 6.371356 | 0.009224 |
|  |  | Gm43519 | 6.181198 | 0.0219 |
|  |  | Foxn4 | 6.181198 | 0.0219 |
|  |  | 3010003L21Rik | 6.181198 | 0.0219 |
|  |  | Tshz3 | 6.181198 | 0.0219 |
|  |  | Gm48453 | 6.181198 | 0.0219 |
|  |  | Prkg1 | 6.181198 | 0.0219 |
|  |  | Cacnb4 | 5.962116 | 0.034783 |
|  |  | Lrp8os2 | 5.962116 | 0.034783 |
|  |  | Slc13a4 | -5.9102 | 0.034783 |
|  |  | Ccdc159 | -5.9102 | 0.034783 |
|  |  | Igf2bp1 | -5.9102 | 0.034783 |
|  |  | Gm49376 | -5.9102 | 0.034783 |
|  |  | Gm49502 | -5.9102 | 0.034783 |
|  |  | Slc2a5 | -6.05981 | 0.0219 |
|  |  | Gm26809 | -6.05981 | 0.0219 |
|  |  | Tnnt3 | -6.05981 | 0.0219 |
|  |  | Gm12454 | -6.19534 | 0.014079 |
|  |  | Spata33 | -6.19534 | 0.014079 |
|  |  | Letm2 | -6.31923 | 0.009224 |
|  |  | 4933430I17Rik | -6.53904 | 0.004166 |
|  |  | Ntng2 | -6.72974 | 0.002864 |
|  |  | Gm26444 | -7.3687 | 0.000218 |
|  |  | Gm22317 | -8.36942 | 1.20E-18 |

RNA Sequencing analysis showing the top 30 genes for each subset (top 15 up-regulated, top 15 down-regulated), sorted by fold change. log2(fold change) and p-value, up- regulated and down- regulated in each subset upon interaction with *C. neoformans* compared to each subset incubated alone. AM = Alveolar macrophages, IM = Interstitial macrophages, Ly6c- =Ly6c- monocyte-like macrophages, Ly6c+ = Ly6c+ monocyte-like macrophages, CD11b+ = CD11b+ DCs, CD103+ = CD103+ DCs.
